# A natural histone H2A variant lacking the Bub1 phosphorylation site and regulated depletion of centromeric histone CENP-A foster evolvability in *Candida albicans*

**DOI:** 10.1371/journal.pbio.3000331

**Published:** 2019-06-21

**Authors:** Cedric A. Brimacombe, Jordan E. Burke, Jahan-Yar Parsa, Sandra Catania, Teresa R. O’Meara, Jessica N. Witchley, Laura S. Burrack, Hiten D. Madhani, Suzanne M. Noble

**Affiliations:** 1 Department of Microbiology and Immunology, University of California, San Francisco, San Francisco, California, United States of America; 2 Department of Biochemistry and Biophysics, University of California, San Francisco, San Francisco, California, United States of America; 3 Department of Biology, Gustavus Adolphus College, Saint Peter, Minnesota, United States of America; 4 Chan-Zuckerberg Biohub, San Francisco, California, United States of America; 5 Department of Medicine, Division of Infectious Diseases, University of California, San Francisco, San Francisco, California, United States of America; Dana-Farber Cancer Institute, UNITED STATES

## Abstract

Eukaryotes have evolved elaborate mechanisms to ensure that chromosomes segregate with high fidelity during mitosis and meiosis, and yet specific aneuploidies can be adaptive during environmental stress. Here, we identify a chromatin-based system required for inducible aneuploidy in a human pathogen. *Candida albicans* utilizes chromosome missegregation to acquire tolerance to antifungal drugs and for nonmeiotic ploidy reduction after mating. We discovered that the ancestor of *C*. *albicans* and 2 related pathogens evolved a variant of histone 2A (H2A) that lacks the conserved phosphorylation site for kinetochore-associated Bub1 kinase, a key regulator of chromosome segregation. Using engineered strains, we show that the relative gene dosage of this variant versus canonical H2A controls the fidelity of chromosome segregation and the rate of acquisition of tolerance to antifungal drugs via aneuploidy. Furthermore, whole-genome chromatin precipitation analysis reveals that Centromere Protein A/ Centromeric Histone H3-like Protein (CENP-A/Cse4), a centromeric histone H3 variant that forms the platform of the eukaryotic kinetochore, is depleted from tetraploid-mating products relative to diploid parents and is virtually eliminated from cells exposed to aneuploidy-promoting cues. We conclude that genetically programmed and environmentally induced changes in chromatin can confer the capacity for enhanced evolvability via chromosome missegregation.

## Abstract 2

The pathogenic fungus Candida albicans uses a variant of histone H2A and regulated depletion of the centromeric histone CENP-A to control the stability of its chromosomes.

## Introduction

Aneuploidy is increasingly recognized as an important form of natural variation in eukaryotes. Chromosome segregation during mitosis and meiosis is generally accurate, but environmental stress has been associated with chromosome instability in multiple species [1, 2]. Here, we investigate the molecular basis for stress- and ploidy-associated genome instability in the diploid (2n) yeast *C*. *albicans*. This organism is the most common agent of fungal disease in humans but primarily exists as a component of mammalian gut, skin, and genitourinary microbiota [3, 4]. The ability of *C*. *albicans* to thrive as both a commensal and pathogen in diverse host niches has been attributed to its ability to transition among multiple specialized cell types [5], as well as its remarkable genomic plasticity and tolerance of aneuploidy [2]. Aneuploidy is common in *C*. *albicans* and is often a response to toxic stress [6]. For example, strains that exhibit resistance to the antifungal drug, fluconazole, frequently carry a duplication of the left arm of Chromosome 5 [7–9], thereby increasing the copy number of the drug target as well as drug efflux pumps. The mechanisms of chromosome loss and aneuploidy generation in *C*. *albicans* are not well established; however, as described below, there is evidence supporting the involvement of tetraploid (4n) intermediates.

*C*. *albicans* tetraploid strains may be generated experimentally by exposure to fluconazole [10] or by mating, wherein diploid *MTL*a and *MTL*α cells mate to form tetraploid (4n) *MTL*a/α progeny [11, 12]. Tetraploid strains generally return to the diploid state either gradually, under standard in vitro conditions, or abruptly, on certain types of media, by a process of random chromosome loss [13, 14]. This unusual “parasexual cycle” is shared by *C*. *albicans* and the related pathogenic species *Candida tropicalis* [15] and *Candida dubliniensis* [16]. In other ascomycetous fungi such as *Saccharomyces cerevisiae*, haploid a or α cells mate to produce diploid a/α progeny (reviewed in [17]), which subsequently undergo meiosis to return to the haploid state. The mechanisms controlling random chromosome loss in *C*. *albicans* have remained elusive.

**Fig 1:**
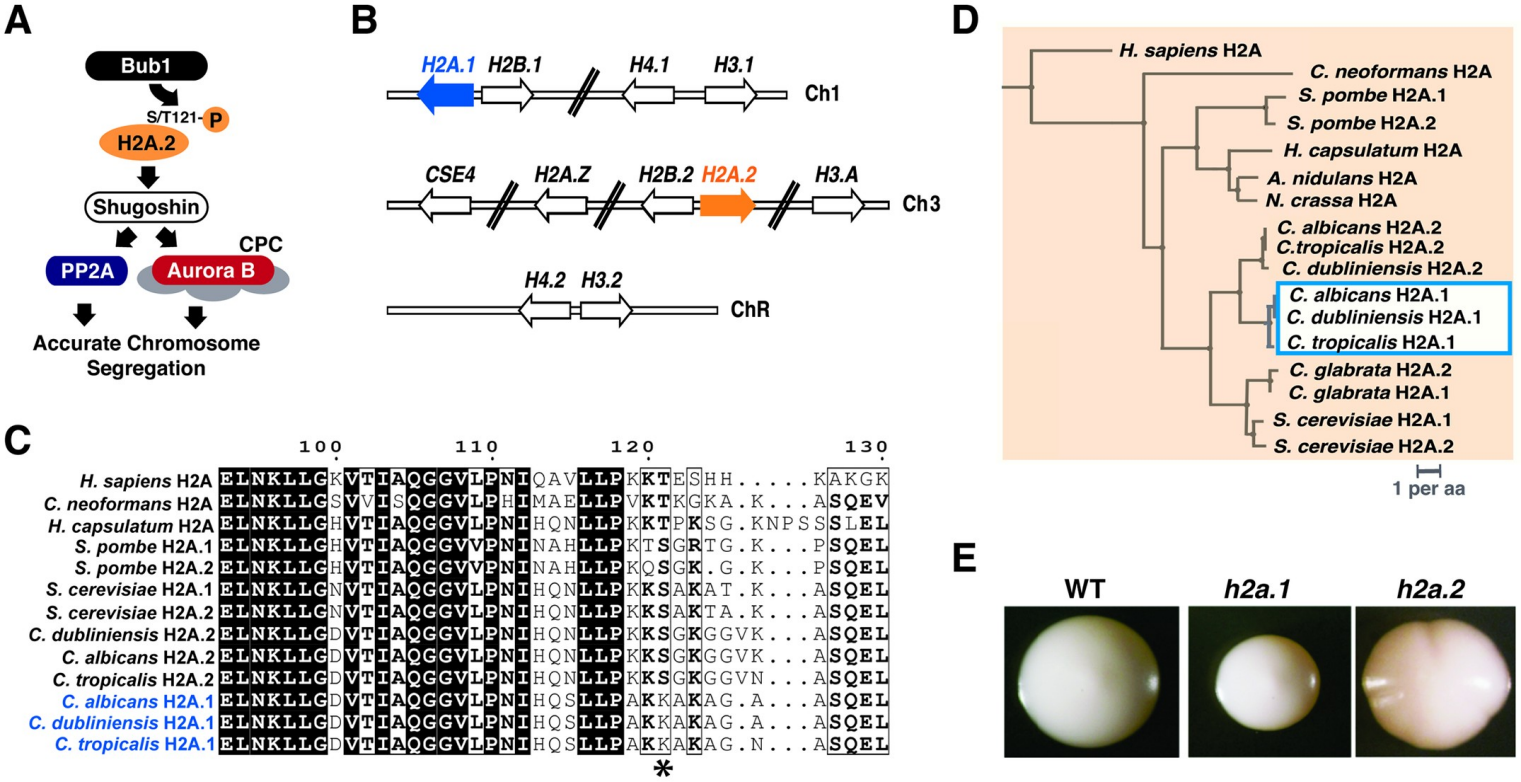
*C*. *albicans* encodes 2 alleles of histone H2A that differ in C-terminal amino acid sequence. (A) Schematic of Bub1–H2A.1–Sgo1 interactions in most eukaryotes. *C*. *albicans* retains orthologs of Bub1 (orf19.2678) and Sgo1 (orf19.3550). (B) *C*. *albicans* histone genes are distributed over 3 chromosomes. *H2A*.*1* (orf19.1051), *H2B*.*1* (orf19.1052), *H4*.*1* (orf19.1061), and *H3*.*1* (orf19.1059) occur on Chromosome 1; *CSE4* (orf19.6163), *H2A*.*Z* (orf19.327), *H2B*.*2* (orf19.6924), *H2A*.*2* (orf19.6925), and *H3*.*A* (orf19.6791) are on Chromosome 3; and *H4*.*2* (orf19.1854) and *H3*.*2* (orf19.1853) are on Chromosome R, which also contains rRNA genes. (C) *C*. *albicans*, *C*. *dubliniensis*, and *C*. *tropicalis* evolved divergent alleles of H2A. Among these 3 yeasts, *H2A*.*1* (blue shading) and *H2A*.*2* (orange shading) are more similar to orthologs in the 2 other species than to the H2A paralog in their own species. Scale bar designates substitutions per amino acid. (D) Amino acid differences between the H2A paralogs of *C*. *albicans*, *C*. *dubliniensis*, and *C*. *tropicalis* (shown in S1 Fig) localize to the C terminus and include replacement of the highly conserved S/T121 residue with lysine (asterisk). (E) Deletion of *H2A*.*2* but not *H2A*.*1* produces a colony sectoring phenotype. WT and homozygous deletion mutants were propagated on YEPD medium and incubated at 30 °C. CPC, Chromosomal Passenger Complex; H2A, Histone 2A; PP2A, Protein Phosphatase 2A; Sgo1, Shugoshin 1; S/T121, serine or threonine at position 121; WT, wild type; YEPD, yeast extract peptone dextrose.

From humans to yeast, chromosome segregation is tightly regulated by a network of conserved checkpoint proteins. Regulated processes include the correction of erroneous microtubule attachment to sister chromatids and protection of centromeric cohesion during mitosis [18]. A central regulator is Bub1 kinase, which phosphorylates histone H2A on a highly conserved serine or threonine at position 121 (S/T121; Fig 1A). This mark subsequently recruits shugoshins [19, 20], which themselves recruit protein phosphatase 2A (PP2A) and the chromosomal passenger complex (CPC), containing Aurora B kinase. PP2A protects centromeric cohesin from degradation and promotes bi-orientation of sister chromatids prior to segregation [21], whereas Aurora B ensures correct microtubule–kinetochore attachment prior to anaphase.

## Results

A search for homologs of Bub1, Shugoshin 1 (Sgo1), and H2A revealed that *C*. *albicans* contains single orthologs of Bub1 and Sgo1 (Fig 1A), as well as 2 homologous core H2A genes paired with divergently transcribed H2B genes, located on Chromosomes 1 and 3, respectively (Fig 1B). We hereafter denote the H2A gene on Chromosome 1 “*H2A*.*1*,” and that on Chromosome 3 “*H2A*.*2*.” The primary amino acid sequences of H2A.1 and H2A.2 differ in the C-terminal region, most notably with the replacement of serine 121 (S121) with a lysine (K121) in H2A.1 but not in H2A.2 (Fig 1C and S1 Fig). A phylogeny of core H2A proteins revealed that the K121-associated allele (H2A.1) also occurs in *C*. *tropicalis* and *C*. *dubliniensis* (Fig 1D), 2 closely related yeast species that share a similar parasexual cycle with *C*. *albicans*. Because mutation of S/T121 results in chromosome instability in other organisms [19], we hypothesized that deletion of *H2A*.*2* but not *H2A*.*1* would produce the same phenotype in *C*. *albicans* and that the evolution of an H2A variant that is resistant to phosphorylation by Bub1 might offer a mechanistic explanation for the chromosome changes observed during the parasexual cycle and upon exposure to aneuploidy-inducing stresses.

To test this hypothesis, we generated knockouts of *H2A*.*1* and *H2A*.*2*, as well as the adjacent *H2B* genes as controls (Fig 1E and S2 Fig). These experiments revealed that deletion of either *H2A*.*1* or *H2B*.*1* results in duplication of Chromosome 3, on which the *H2A*.*2*–*H2B*.*2* locus is located; however, the converse is not true when *H2A*.*2* or *H2B*.*2* is deleted (S2 Fig, S1 Table). An analogous duplication of the *H2A–H2B* locus occurs in *S*. *cerevisiae* when 1 set of these loci is deleted, although in this case, duplication occurs via the formation of a small circular episome rather than an entire chromosome [22]; a similar phenomenon occurs with reduction in histone H4 gene dosage in *C*. *albicans* [23].

Because deletion of *H2A*.*1* results in duplication of Chromosome 3, we used an alternative strategy to determine the phenotype of isogenic strains that lack either H2A gene. As shown in S3A Fig, “open reading frame (ORF) swapped” strains were constructed in which both copies of the *H2A*.*1* ORF are precisely replaced with the *H2A*.*2* ORF and vice versa. The resultant strains contain either *H2A*.*1* or *H2A*.*2* at all 4 core H2A loci and thus represent null mutants of the other histone gene. Neither ORF-swapped strain contains aneuploidies, and measurements of genomic copy number and mRNA levels confirmed correct ORF replacement and transcription in the ORF-swapped strains (S3A–S3C Fig). For clarity, we hereafter refer to experimental strains based on the identities of each H2A ORF on Chromosomes 1 and 3 using the following nomenclature: [## (Chromosome 1),## (Chromosome3)], with “1” representing *H2A*.*1*, “2” representing *H2A*.*2*, and “-”representing the absence of an ORF. Thus, a diploid strain containing 4 copies of *H2A*.*1* would be denoted [11,11] (see example in S3A Fig).

**Fig 2:**
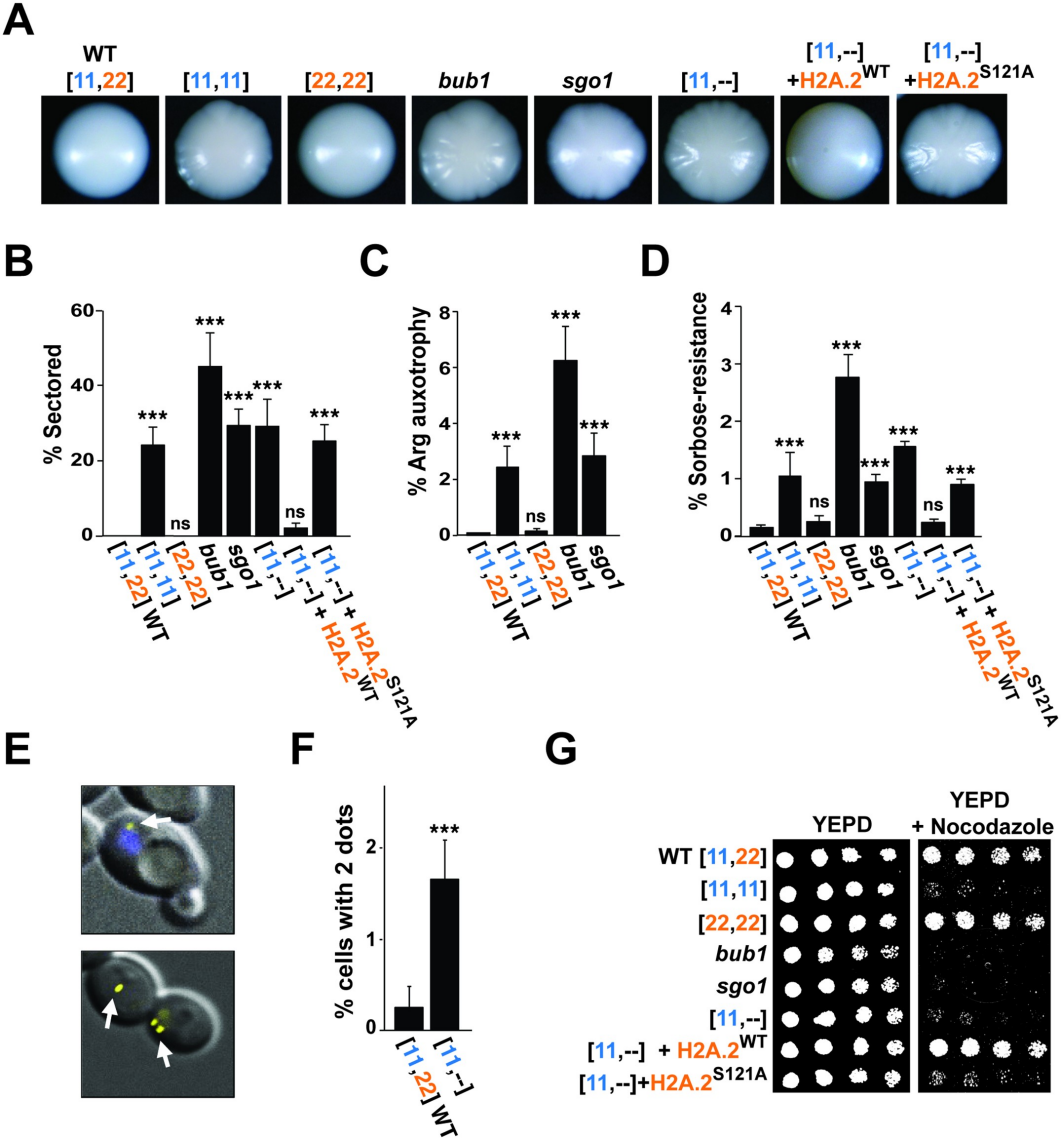
A *C*. *albicans* mutant lacking *H2A*.*2* phenocopies spindle-checkpoint mutants. (A and B) Deletion of *H2A*.*2*, *BUB1*, or *SGO1*, or replacement of *H2A*.*2* with an *H2A*.*2* S121A mutation, results in similar colony sectoring phenotypes. Colony morphology (A) and percent of colony sectoring (B) are shown for WT *C*. *albicans*, the [11,11] strain that lacks *H2A*.*2*, the [22,22] strain that lacks *H2A*.*1*, knockouts of the spindle-checkpoint genes, *BUB1* and *SGO1*, and [11,--], [11,--]+*H2A*.*2*^WT^, and [11,--]+*H2A*.*2*^S121A^ strains. (C, D, E, and F) Mutants lacking *H2A*.*2*, *BUB1*, or *SGO1* display increased aneuploidy. (C) Percent of colonies that have lost the ability to grow on arginine-deficient medium, relative to growth on YEPD. Strains were plated on arginine-deficient medium or YEPD after 4 days on presporulation medium. Because these *ARG4/*Δ strains contain the *ARG4* gene on only 1 copy of Chromosome 7, loss of that copy renders cells auxotrophic for arginine. (D) Percent of colonies that grow on sorbose-containing medium, relative to growth on YEPD. Because cells with both copies of Chromosome 5 are killed by sorbose, this medium selects for cells that have lost 1 copy of the chromosome. (E, top panel) Superimposed fluorescence and phase microscopy of QMY85, which contains approximately 120 tandem copies of the *tet* operator on one of its 2 Chromosome 5 homologs, as well as the Tet repressor fused to YFP. A yellow spot marks the tagged copy of Chromosome 5 (white arrow), and the nucleus is stained with DAPI (blue arrow). (Bottom panel) Example of neighboring cells that contain 1 spot (normal) or 2 spots (indicating an extra copy of Chromosome 5). (F) Percent of cells with 2 spots, counting only unbudded cells. (G) Sensitivity to the microtubule poison nocodazole (50 μM) shown by serial dilution. Please see S1 Data for the numerical values summarized in (B, C, D, and F). prespo, presporulation; WT, wild type; YEPD, yeast extract peptone dextrose; YFP, Yellow Fluorescent Protein.

We tested the hypothesis that *H2A*.*2* but not *H2A*.*1* is required for chromosome stability by comparing the phenotypes of strains lacking either histone gene. On standard growth medium, a strain lacking *H2A*.*1* ([22,22]) is indistinguishable from wild type (WT); however, a strain lacking *H2A*.*2* ([11,11]) exhibits sectoring in roughly 25% of colonies (Fig 2A and 2B). Colony sectoring is often symptomatic of chromosome instability, with defects in colony margins reflecting clones of inviable cells. Consistent with this interpretation, deletion mutants affecting the upstream (Bub1) and downstream (Sgo1) spindle-assembly checkpoint components also exhibit sectoring phenotypes (Fig 2A and 2B). An *sgo1* mutant sectors to a similar degree as the [11,11] strain, whereas *bub1* has an even stronger phenotype, likely reflecting additional checkpoint functions of Bub1 [18, 19]. We queried the role of the S121 residue of H2A.2 on the phenotype by testing the ability of WT *H2A*.*2* versus a mutant differing by an S121A substitution to suppress colony sectoring. When restored to the genome of the *h2a*.*2* strain (i.e., [11,--]), WT *H2A*.*2* reduces sectoring to 2% of colonies, but the S121A variant has no effect (Fig 2A and 2B). These results suggest that increased colony sectoring is linked to H2A alleles that are not substrates for Bub1 kinase.

We next assessed chromosome stability in these strains by introducing heterozygosity for the auxotrophic marker *ARG4* (located on Chromosome 7), followed by testing for marker loss over time. Compared with WT or the [22,22] mutant, strains that lack H2A.2, Bub1, or Sgo1 exhibit highly elevated rates of marker loss that correlate with their colony sectoring phenotypes (compare Fig 2C and 2B). Similar results were obtained when cells were evaluated on sorbose-containing medium, which selects for cells that have lost a single copy of Chromosome 5 (Fig 2D); in this assay, restoration of 1 allele of WT *H2A*.*2* to the [11,--] strain reduced the number of colonies that grow on sorbose, whereas the S121A-containing variant again had no effect (Fig 2D). In a reciprocal experiment, we tested for chromosome gain events using an *h2a*.*2* mutant ([11,--]), in which 1 copy of Chromosome 5 can be visualized as a fluorescent dot, produced by the binding of a Tetracycline Repressor Protein-Yellow Fluorescent Protein (TetR-YFP) fusion protein to >100 tandem copies of the *tet* operator (Fig 2E). As shown in Fig 2F, the frequency of unbudded cells that contain 2 nuclear dots is approximately 6 times higher in the *h2a*.*2* mutant than in WT *C*. *albicans*. Finally, all strains that lack WT H2A.2—including an *h2a*.*2* strain that contains the S121A variant—as well as *bub1* and *sgo1* are hypersensitive to the microtubule-destabilizing drug nocodazole (Fig 2G), as would be expected for mutants affecting the spindle-assembly complex [19]. The nocodazole-hypersensitive strains are also hypersensitive to ultraviolet radiation (S4 Fig), consistent with an additional role for *C*. *albicans* H2A S121 in DNA repair as has been observed in other yeasts [19]. These results support our strong expectation, based on studies in other organisms, that H2A.2 but not H2A.1 acts as a substrate of the Bub1 kinase to recruit shugoshin to centromeric chromatin and thereby to ensure faithful chromosome segregation (Fig 1A). However, they raise the question of why *C*. *albicans* and closely related yeasts would maintain a gene encoding a defective histone H2A.

**Fig 3:**
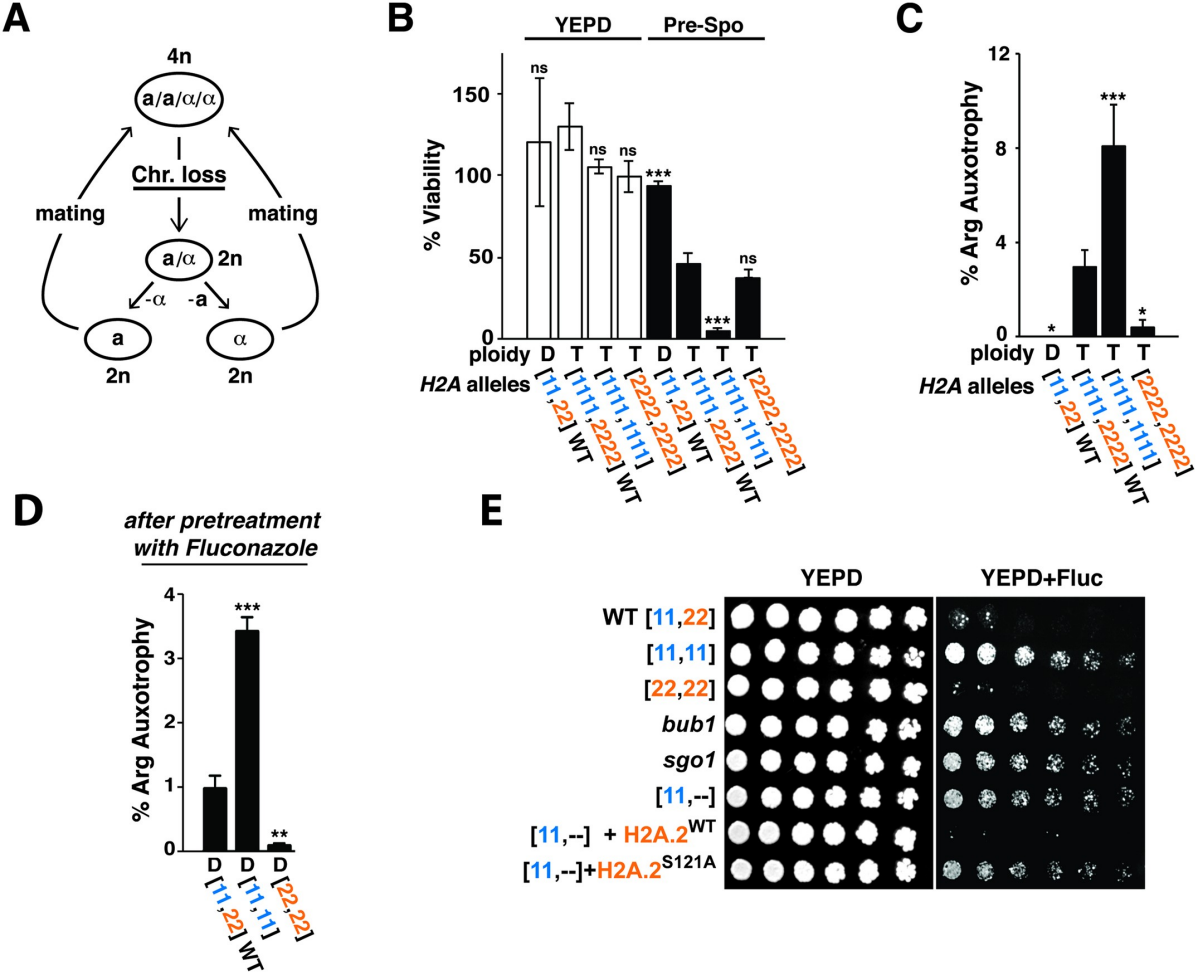
H2A.1 and H2A.2 play opposing roles in chromosome stability in tetraploid cells and in the acquisition of tolerance to fluconazole. (A) Cartoon of the *C*. *albicans* parasexual cycle, which consists of mating between diploid a and α cells to form tetraploid a/α cells, followed by ploidy reduction by concerted chromosome loss rather than meiosis, as occurs in other eukaryotes. (B) Viability of WT diploid, WT tetraploid, or tetraploid cells containing only H2A.1 ([1111,1111]) or H2A.2 ([2222,2222]) on YEPD (left) versus prespo medium (right). Plated cells were propagated for 4 days at 37 °C, and survival was calculated as the ratio of colonies on each medium to the estimated number of plated cells determined using a hemocytometer. Error bars represent standard deviation. Significant differences from the tetraploid WT strain were determined using one-way ANOVA with Dunnett’s correction for multiple comparisons; *p < 0.05, **p < 0.01, ***p < 0.001. (C) Chromosome stability of *ARG4/arg4*Δ WT diploid, WT tetraploid, and derivative strains on prespo medium after 4 days, as measured by growth on SC-Arg medium, indicative of the loss of 1 copy of Chromosome 7. (D) Chromosome stability of *ARG4/arg4*Δ WT diploid and derivative strains after 3 days of pregrowth in YEPD containing 1 μg/ml fluconazole. (E) Serial dilutions of *C*. *albicans* diploid strains (WT = [11,22], [11,11], [22,22], *bub1*, *sgo1*, *h2a*.*2* = [11,--], *h2a*.*2*+*H2A*.*2*^WT^, and *h2a*.*2*+*H2A*.*2*^S121A^) plated onto YEPD or YEPD + fluconazole (10 μg/ml). Please see S1 Data for the numerical values summarized in (B, C, and D). Chr, Chromosome; Fluc, Fluconazole; H2A, Histone 2A; n.s., nonsignificant; prespo, presporulation; SC-Arg, Synthetic complete lacking arginine; WT, wild type; YEPD, yeast extract peptone dextrose.

Based on the observations that (1) reduced H2A.2 levels result in chromosome instability in diploid cells (Fig 2C, 2D and 2F), (2) *C*. *albicans* tetraploid cells exhibit an enhanced rate of random chromosome loss (Fig 3A), and (3) the H2A.1 variant is maintained by 3 closely related yeast species that undergo parasexual mating (Fig 1B and 1C), we posited that the presence of H2A.1 may promote ploidy reduction in tetraploid cells. To test this hypothesis, we compared the stability of chromosomes in WT tetraploid cells ([1111,2222]) versus tetraploids that contain only H2A.1 ([1111,1111]) or only H2A.2 ([2222,2222]); ploidy of the strains was verified by flow cytometry (S5A and S5B Fig). Viability and genomic marker loss were assayed on presporulation (prespo) medium, which strongly promotes ploidy reduction in tetraploid cells [13]. Note that concerted chromosome loss is associated with reduced cell viability [13]. We observed that, after 4 days of propagation on prespo medium, the viability of WT and [2222,2222] tetraploids was between approximately 40% and 50%, whereas that of the [1111,1111] tetraploid was more drastically reduced to only approximately 4% (Fig 3B); by comparison, there were no viability differences among the strains when propagated on standard yeast extract peptone dextrose (YEPD) medium (Fig 3B). The decreased viability of the [1111,1111] tetraploid strain that lacks canonical H2A.2 was associated with a significantly elevated rate of chromosomal marker loss compared to the WT tetraploid strain (Fig 3C). By contrast, the [2222,2222] tetraploid strain that lacks the variant histone H2A.1 exhibited a significantly reduced rate of marker loss (Fig 3C). These results suggest that both *H2A* homologs contribute to the net stability of chromosomes in WT tetraploid cells, with H2A.1 promoting chromosome-loss and -gain events and H2A.2 promoting maintenance of an eu-tetraploid state. The chromosome stabilizing function of H2A.2 is further supported by the enhanced colony sectoring (S5C Fig) and cell death (prespo medium; Fig 3B) phenotypes displayed by tetraploid strains that lack this homolog.

Our observation of a role for H2A.1 in destabilizing chromosomes in tetraploid cells (Fig 3C) raised the question of why similar activity was not detected in diploid cells (Fig 2C and 2D). In other words, why doesn’t a [22,22] ORF-swap strain that lacks H2A.1 display lower rates of chromosome loss than a WT [11,22] diploid? We hypothesized that the low rate of chromosome loss displayed by WT diploids under standard laboratory conditions (Fig 3C) impedes the ability to detect increases in chromosome stability. To test this idea, we exposed *ARG4*/Δ diploid strains to low-dose (1 μg/ml) fluconazole, a drug that promotes aneuploidy in this species [10], before remeasuring rates of arginine auxotrophy (loss of Chromosome 7). Whereas auxotrophy was virtually undetectable in a WT *ARG4*/Δ diploid when propagated under standard conditions (Fig 3C), the same strain exhibited approximately 1% arginine auxotrophy following exposure to fluconazole (Fig 3D). By comparison, the [11,11] ORF-swap strain that lacks canonical H2A.2 exhibited a further increase to approximately 3.5% arginine auxotrophy (*p* < 0.001 compared to WT, one-way ANOVA), whereas the [22,22] strain that lacks noncanonical H2A.1 maintained a low rate of <0.1% despite exposure to fluconazole (*p* < 0.01 compared to WT). Quantitative polymerase chain reaction (qPCR) analysis of selected arginine auxotrophs and prototrophs confirmed that auxotrophy usually results from loss of Chromosome 7 (S2 Table). Together, these data indicate that *C*. *albicans* H2A.1 promotes chromosome instability in diploid as well as tetraploid strains.

Because aneuploidy is frequently associated with resistance to fluconazole [10], the most commonly prescribed antifungal drug, we tested *C*. *albicans* diploid strains that display different rates of chromosome instability for sensitivity to this drug. Dilution series were prepared from WT, the [11,11] and [22,22] ORF-swap strains, *bub1*, *sgo1*, an *h2a*.*2* null mutant ([11,--]), and *h2a*.*2* complemented with either *H2A*.*2*^WT^ or *H2A*.*2*^S121A^, and the strains were spotted onto YEPD versus YEPD plus 10 μg/ml fluconazole. As shown in Fig 3E, strains with increased chromosome instability ([11,11], *bub1*, *sgo1*, *h2a*.*2*, and *h2a*.*2*+*H2A*.*2*^S121A^) exhibited enhanced survival compared to strains with lower chromosome instability (WT, [22,22], or *h2a*.*2*+*H2A*.*2*^WT^). Of note, because WT exhibits very low tolerance to fluconazole in this assay, it was not possible to determine whether tolerance is further diminished in the [22,22] strain that lacks H2A.1. Overall, these results demonstrate that chromosome instability can provide a selective advantage under certain kinds of stress.

**Fig 4:**
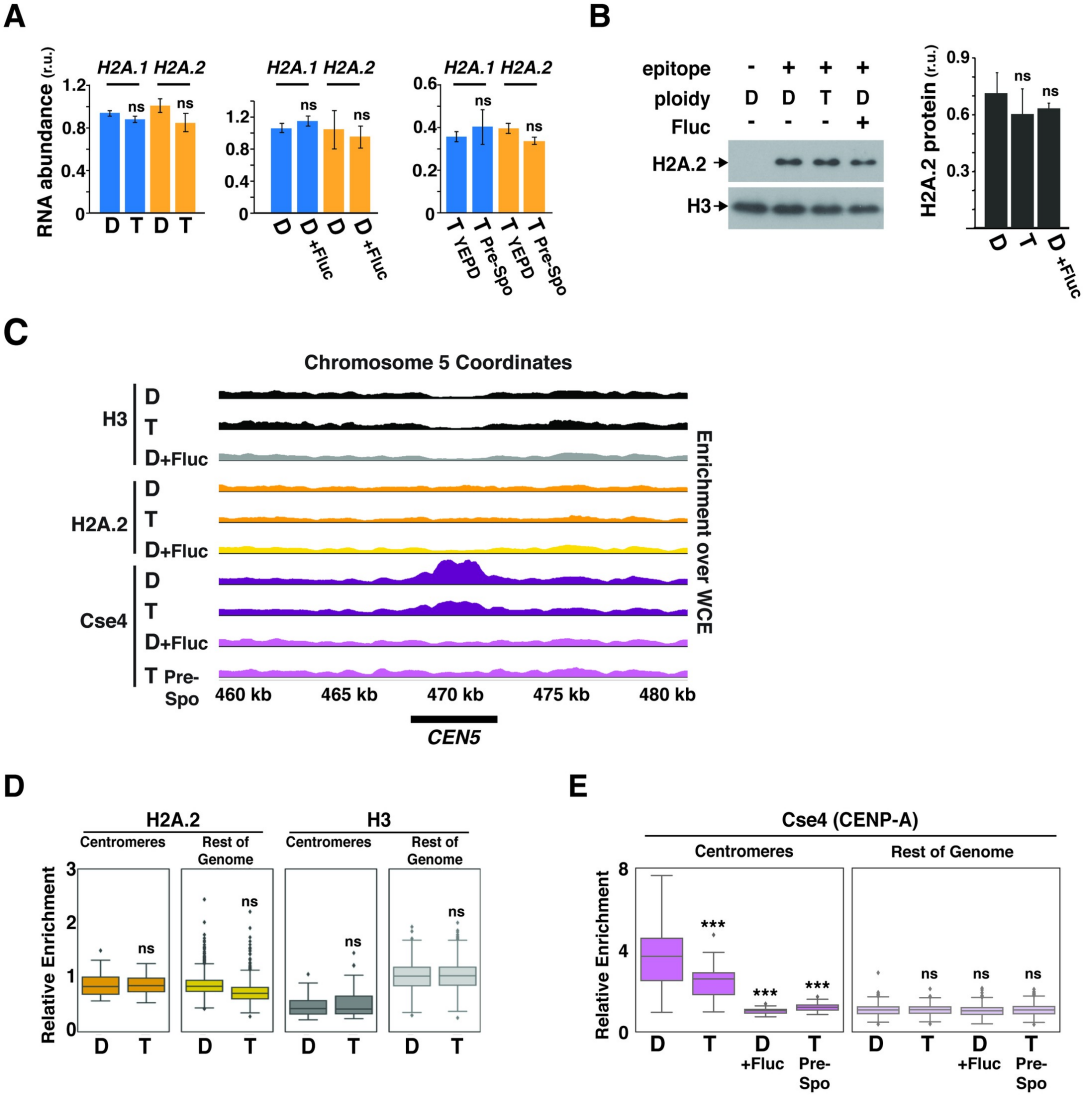
Impact of ploidy and environment on histone expression and deposition. (A) RT-qPCR analysis of expression of *H2A*.*1* and *H2A*.*2* in the indicated genotypes and conditions. “D” represents a WT [11,22] diploid strain and “T” a WT [1111,2222] tetraploid strain. The average results for 3 biological replicates are presented after normalization to H3 mRNA, along with the standard deviation. There were no significant differences by one-way ANOVA. (B) Immunoblot of levels of H2A.2-FLAG in the indicated genotypes and conditions. Quantification using a LI-COR Odyssey imaging system is shown on the right. There were no significant differences by one-way ANOVA. (C) ChIP-seq analysis. Plots of bedgraphs of normalized read densities are shown for the indicated genotypes and conditions. Data represent the results of 1 replicate of each sample for *CEN5*. Data for all centromeres are shown in S6 Fig, and scatterplots of replicate data are shown in S7 Fig. (D and E) Boxplots showing normalized ChIP-seq enrichments for H2A.2 (D), H3 (D), and Cse4/CENP-A (E) (relative to the WCE sample; see Materials and methods) of nonoverlapping 5 kb tiles of the *C*. *albicans* genome separated into those overlapping with annotated centromeres versus the remainder of the genome. *p*-Values were determined using the Mann-Whitney U test. Cse4/CENP-A association with centromeres was significantly different in diploids versus tetraploids (*p* < 1 × 10^−5^), diploids propagated in YEPD versus YEPD plus fluconazole (*p* < 1 × 10^−15^), and tetraploids propagated in YEPD versus prespo (*p* < 1 × 10^−13^). Please see S1 Data for the numerical values summarized in (A). ChIP-seq data are available from the NCBI GEO website (accession number GSE122037). CENP-A, Centromere Protein A; ChIP-seq, chromatin immunoprecipitation with massively parallel DNA sequencing; Cse4, Centromeric Histone H3-like Protein; Fluc, fluconazole; GEO, Gene Expression Omnibus; H2A, Histone 2A; NCBI, National Center For Biotechnology Information; n.s., not significant; prespo, presporulation; RT-qPCR, reverse-transcription quantitative PCR; r.u., relative units; WCE, whole-cell extract; WT, wild type; YEPD, yeast extract peptone dextrose.

The presence of H2A.1 provides a mechanistic explanation for the enhanced basal rate of chromosome instability in *C*. *albicans*; however, it does not explain why chromosomes are destabilized in certain ploidy- and stress-dependent contexts. In other words, why are chromosome gain and loss events more frequent in tetraploid cells compared to diploid cells (Fig 3C), in tetraploid cells propagated in prespo medium compared to YEPD (Fig 3B and [13]), and in diploid cells treated with fluconazole compared to untreated cells [10]? Given the contrasting phenotypes of strains genetically engineered to express only H2A.1 or H2A.2, we initially hypothesized that the relative abundance of H2A.1 versus H2A.2 mRNA or protein might be regulated to modulate the stability of chromosomes in a context-dependent fashion. However, reverse transcription with quantitative PCR (RT-qPCR) analysis of *H2A*.*1* and *H2A*.*2* mRNA levels revealed no significant differences between any of these cell types or conditions (*p* > 0.05, one-way ANOVA, Fig 4A). Likewise, the abundance of an epitope-tagged H2A.2 fusion protein (H2A.2-FLAG) did not appear to vary with ploidy or exposure to fluconazole (*p* > 0.05, one-way ANOVA, Fig 4B). Notably, epitope-tagged H2A.1 could not be obtained despite multiple attempts. Having failed to detect regulation of H2A mRNA or protein levels, we next hypothesized that the localization of H2A on chromosomes might be a target of regulation. For example, if canonical H2A.2 were shunted away from centromeres and pericentric DNA under chromosome-destabilizing conditions, then only nonphosphorylatable H2A.1-containing nucleosomes would be available to interact with kinetochore components. However, Chromatin immunoprecipitation with massively parallel DNA sequencing (ChIP-seq) analysis of H2A.2-FLAG did not reveal statistically significant differences in localization between tetraploid versus diploid cells, or in diploid cells propagated in YEPD versus YEPD plus 10 μg/ml fluconazole (*p* > 0.05 for all pairwise comparison of replicates, Mann-Whitney U tests; Fig 4C and 4D, S6 and S7 Figs). Control ChIP-seq experiments performed with anti-H3 antibodies revealed depletion at centromeres in all cell types and conditions (Fig 3C and 3D, S6 and S7 Figs), as expected if canonical histone H3 is replaced at these locations by the centromere-specific histone H3, Cse4 [24] (CENP-A in other organisms [25]).

Extensive biochemical and genetic data support a close interaction between H2A and Cse4/CENP-A within centromeric nucleosomes in *S*. *cerevisiae* [26–30]. Given that Cse4/CENP-A forms the platform for assembly of eukaryotic kinetochores [25], we hypothesized that changes in its association with centromeric chromatin might synergize with the presence of H2A.1-containing nucleosomes to increase chromosome instability. We therefore investigated a potential role for Cse4/CENP-A in ploidy-dependent regulation, using polyclonal antibodies against the centromere-specific histone H3 to perform ChIP-seq on extracts prepared from WT diploid versus tetraploid cells. Analysis of replicate samples revealed enrichment of Cse4 at all 7 centromeres (Chromosome R that contains tandem repeats of ribosomal DNA was excluded from analysis), as expected; however, the level of Cse4 was significantly reduced in tetraploid cells compared to diploids (*p* < 1 × 10^−5^ for all 4 pairwise replicate comparisons; Mann-Whitney U tests; Fig 4C and 4E, S6 and S7 Figs). To determine whether Cse4/CENP-A deposition is influenced by in vitro conditions associated with enhanced chromatin instability [10, 13], we repeated the analysis on extracts prepared from diploid cells exposed to 10 μg/ml fluconazole and tetraploid cells propagated in prespo medium. Under chromosome-destabilizing conditions, we observed an even more striking depletion of Cse4 at centromeres (Fig 4C and 4E, S6 and S7 Figs; *p* < 1 × 10^−15^ for all 4 pairwise replicate comparisons of diploid cells and *p* < 1 × 10^−13^ for equivalent comparisons of tetraploid cells; Mann-Whitney U tests).

These data indicate that at least 2 pathways promote chromosome instability in *C*. *albicans*. A newly evolved H2A lacking the Bub1 phosphorylation site promotes chromosome instability under standard conditions by generating nucleosomes that are ineffective at recruiting shugoshin. We hypothesize that the presence of variant H2A.1 in centromeric nucleosomes sensitizes cells to changes in other centromere and kinetochore components. Thus, the graded depletion of Cse4/CENP-A from centromeres in cells with increased ploidy (moderate effect) or in stressful environments (strong effect) produces further increases in chromatin instability and aneuploidy.

## Discussion

Chromosome instability is proposed to play key roles in *C*. *albicans* cell biology, including reduction of chromosome number after mating and adaptation to drug challenges. Although substantial evidence supports the ability of environmental perturbations to trigger chromosome instability in this organism [6, 10, 13, 31], the underlying molecular requirements have remained obscure. We discovered that *C*. *albicans* and the related human pathogens *C*. *tropicalis* and *C*. *dubliniensis* have evolved a variant histone H2A protein, H2A.1, that is defective in chromosome segregation because of loss of an otherwise universally conserved phosphorylation site of the Bub1 kinase. We show that H2A.1 promotes ploidy modulation in standard diploid cells as well as tetraploid mating products and enhances the acquisition of tolerance to fluconazole. Under aneuploidy-inducing growth conditions, strains that lack the noncanonical histone exhibit lower rates of chromosome loss than WT, demonstrating that WT is not tuned for maximal chromosome stability.

Furthermore, our genome-wide analysis of histone localization revealed ploidy- and environment-dependent effects on the level of Cse4/CENP-A at *C*. *albicans* centromeres. Specifically, we observed significant, often dramatic, reductions of this centromeric H3 variant in genotypes and conditions that display increased rates of aneuploidy. Because CENP-A forms the conserved foundation of the eukaryotic kinetochore, these reductions are expected to produce profound defects in kinetochore function. We conclude that at least 2 histone-based mechanisms promote chromosome instability in *C*. *albicans*. These mechanisms for modulating the fidelity of chromosome segregation foster parasexuality and the rapid acquisition of tolerance to drugs and other environmental stresses.

Variation is an essential requirement for evolution of all organisms, and aneuploidy is a surprisingly common form of natural variation. For example, 19% of natural isolates of *S*. *cerevisiae* are aneuploid [32], despite the fact that mitosis is a high-fidelity process in this species, at least under standard laboratory conditions. Although aneuploidy is typically deleterious, specific aneuploidies have been shown to confer selective advantages to organisms ranging from cancer cells [33] to fungi [34] in times of stress. Given that many eukaryotes are subject to severe and unpredictable environmental fluctuations, the capacity for programmed variation through promiscuous chromosome segregation may be more general than currently appreciated.

## Materials and methods

### Media and growth conditions

Cells were propagated in liquid YEPD (1% yeast extract [Becton Dickinson, Franklin Lakes, NJ], 2% peptone [Sigma-Aldrich, St. Louis, MO], 2% dextrose [Sigma-Aldrich, St. Louis, MO], YEPD+2% agar [Becton Dickinson, Franklin Lakes, NJ]), liquid prespo medium (0.8% yeast extract, 0.3% peptone, 10% dextrose), or prespo + 2% agar as previously described by Bennett and colleagues [13]. For selection of nourseothricin-resistant (NatR) transformants, nourseothricin (Nat; Research Products International, Mt. Prospect, IL) was added to YEPD agar to a final concentration of 200 μg/mL. For selection by auxotrophic markers, synthetic defined (SD) + 2% dextrose agar medium without selected amino acids (arginine, arg; histidine, his; or leucine, leu) was used (yeast nitrogen base without amino acids (Becton Dickinson, Franklin Lakes, NJ); all amino acids: Sigma-Aldrich, St. Louis, MO; Homann and colleagues). For colony morphology analyses, cells were propagated on YEPD agar at 30 °C for 5 days. For chromosome stability analyses, cells were propagated on prespo agar medium at 37 °C for 4 days. For mRNA and protein analyses, cells were propagated in YEPD in a 30 °C shaker running at 200 rpm to an OD of approximately 1.0 before harvest for use in subsequent assays. For fluorescence microscopy analyses, cells were grown similarly to those prepared for mRNA analyses.

### Strain construction

A detailed description of strains, all of which are derivatives of the clinical isolate SC5314 [35], is provided in S3 Table. *C*. *albicans* mutant and epitope-tagged strains were created as previously described by Hernday and colleagues [36]. orf19 standard designations of genes newly named in this work include *H2A*.*1* (orf19.1051), *H2B*.*1* (orf19.1052), *H2A*.*2* (orf19.6925), *H2B*.*2* (orf19.6924), *H3*.*1* (orf19.1059), *H4*.*1* (orf19.1061), *H3*.*2* (orf19.1853), *H4*.*2* (orf19.1854), *H3*.*A* (orf19.6791), and *H2A*.*Z* (orf19.327).

#### ORF swapping

All plasmids and primers (synthesized by Integrated DNA Technologies, Coralville, IA) used in strain construction and diagnosis are described in S4 and S5 Tables, respectively. H2A ORF-swapped strains were created using plasmids pSN379 and pSN380. pSN379 contains 300 base pairs (bp) upstream of the *H2A*.*1* ORF fused to *H2A*.*2* ORF (amplified from genomic DNA using primers SNO2811 and SNO2812), the *H2A*.*2* ORF (amplified from genomic DNA using SNO2313 and SNO2314), an *FLP-SAT* cassette [37] (amplified from pSFS2 [37] using SNO2815 and SNO2816), and 300 bp downstream of the *H2A*.*1* ORF (amplified from genomic DNA using SNO2017 and SNO2818), flanked on either side by PmeI restriction sites. pSN380 contains 300 bp upstream of the *H2A*.*2* ORF fused to the *H2A*.*1* ORF (using SNO2819 and SNO2820), the *H2A*.*1* ORF (using SNO2821 and SNO2822), the *FLP-SAT* cassette (using SNO2823 and SNO2824), and 300 bp downstream of the *H2A*.*2* ORF (using SNO2825 and SNO2826), flanked on either side by PmeI restriction sites. These constructs were assembled by homologous recombination in *S*. *cerevisiae* [38]. Plasmids were linearized by digestion with PmeI (New England Biolabs, Ipswich, MA) and transformed into the WT strain SN152, with selection for nourseothricin-resistant (NatR) colonies. Correct integration was verified by colony PCR targeting the 5ʹ and 3ʹ junctions using the following primers: SNO2859 and SNO2867; SNO2862 and SNO2868 for integration of pSN379; SNO2863 and SNO2867; and SNO2866 and SNO2868 for integration of pSN380. Cells were propagated in YEP plus 2% maltose (Thermo Fisher Scientific, Waltham, MA) to permit excision of *FLP-SAT* cassette, with correct excision verified by PCR of the 5ʹ and 3ʹ junctions. Each Nat-sensitive heterozygous strain was then transformed a second time with the same linearized plasmid, again selecting for NatR colonies. Colony PCR was performed to confirm the absence of each ORF, using SNO2735 and SNO2736 for *H2A*.*1* and SNO2549 and SNO2550 for *H2A*.*2*. Colonies that were ORF negative for each opposite histone were validated for correct insertion by PCR across the 5ʹ and 3ʹ junctions. The *FLP-SAT* cassette was subsequently flipped out, with correct excision verified by junction PCR. The resultant strains contained 4 copies of *H2A*.*1* or *H2A*.*2*, with 2 copies in the native locus and 2 copies in the other *H2A* locus, with FRT sites immediately 3ʹ of the stop codon of each gene. The absence of *H2A*.*1* native junctions was verified using SNO2661 and SNO2972 (5ʹ), and SNO2662 and SNO2971 (3ʹ). The absence of *H2A*.*2* native junctions was verified using SNO2547 and SNO2974 (5ʹ) and SNO2603 and SNO2973 (3ʹ).

#### Targeted gene disruption

Homozygous knockout mutants of *H2A*.*1*, *H2B*.*1*, *H2A*.*2*, *H2B*.*2*, *BUB1*, *SGO1*, *and MTL*a were constructed by fusion PCR as previously described [39]. For each strain, a brief description of primers used is as follows: *H2A*.*1* deletion constructs were created using the primers SNO2657–SNO2660, and correct integration was verified using SNO2661, SNO2662, and SNO265–268. Strains were verified as ORF negative using the primers SNO2735 and SNO2736. *H2A*.*2* deletion constructs were created using the primers SNO2543–SNO2546, and correct integration was verified using SNO2547, SNO2603, and SNO265–268. Strains were verified as ORF negative using the primers SNO2549 and SNO2550. *H2B*.*1* deletion constructs were created using the primers SNO2748–SNO2751, and correct integration was verified using SNO2752, SNO2753, and SNO265–268. Strains were verified as ORF negative using the primers SNO2725 and SNO2726. *H2B*.*2* deletion constructs were created using the primers SNO2665–SNO2668, and correct integration was verified using SNO2669, SNO2670, and SNO265–268. Strains were verified as ORF negative using the primers SNO2729 and SNO2730. *BUB1* deletion constructs were created using the primers SNO2999–SNO3002, and correct integration was verified using SNO3003, SNO3004, and SNO265–268. Strains were verified as ORF negative using the primers SNO3005 and SNO3006. *SGO1* deletion constructs were created using the primers SNO3086–SNO3089, and correct integration was verified using SNO3090, SNO3091, and SNO265–268. Strains were verified as ORF negative using the primers SNO3092 and SNO3093. *MTL*a deletion constructs were created using the primers SNO2502–SNO2505. Strains were verified as ORF negative using the primers SNO2358 and SNO2359.

A homozygous knockout of *H2A*.*2* in the TetO-TetR-YFP strain was generated using the plasmid pSN383, with 2 successive rounds of transformation followed by excision of the *FLP-SAT* cassette. pSN383 contains approximately 300 bp upstream of the *H2A*.*2* ORF (amplified from genomic DNA using primers SNO3145–3146), the *FLP-SAT* cassette (amplified from pSFS2 using primers SNO3149–3150), and approximately 300 bp downstream of the *H2A*.*2* ORF (amplified from genomic DNA using primers SNO3147–3148); both sides were flanked by PmeI restriction sites for linearization. Constructs were assembled by homologous recombination in *S*. *cerevisiae* and sequenced. Linearized plasmid was transformed into *C*. *albicans* strain QMY85, and NatR colonies were selected; correct 5ʹ and 3ʹ integration events were confirmed using the primers SNO2543 and SNO1902, and SNO2546 and SNO1903. The FLP-SAT cassette was excised as described above, and a second round of transformation and *FLP-SAT* excision was performed. Strains were verified as ORF negative for *H2A*.*2* using the primers SNO2549–2550.

#### Complementation with H2A.2 and H2A.2 S121A

The *H2A*.*2* gene addback construct was generated by precisely replacing the ORF of a previously used addback construct [40] with the *H2A*.*2* ORF, flanked by approximately 1,000 bp 5ʹ and 3ʹ of the gene, resulting in pSN381. Primers SNO3068 and SNO3071 were used to amplify the backbone of the plasmid, and SNO3068 and SNO3069 were used to amplify the inserted sequence. Products were then treated with DpnI (New England Biolabs, Ipswich, MA), mixed in a 1:1 ratio, and transformed into *Escherichia coli*, followed by sequencing of transformants to verify correct DNA sequence replacement. The S121A mutation was introduced by synthesizing an *H2A*.*2* gene that contains a TCA to GCA codon substitution at this position; the mutant ORF was subsequently swapped into the original addback construct by homologous recombination in *S*. *cerevisiae* [38], generating pSN382 using the same strategy as above, with correct replacement verified by sequencing. Each resultant plasmid contains several hundred bp of genomic sequence immediately upstream of the *LEU2* ORF, followed by the *H2A*.*2* (WT) or *H2A*.*2* (S121A) gene, *C*. *dubliniensis ARG4*, and several hundred bp of genomic sequence immediately downstream of the *LEU2* ORF, all of which are flanked by PmeI restriction sites, identical to previously described addback plasmids [41]. Plasmids were linearized by digestion with PmeI, and transformed into the [2,0] diploid strain, with selection for Arg^+^ transformants. Presence of the ORF and the expected 5ʹ and 3ʹ junctions was verified by colony PCR using the primers SNO2549–2550 (ORF), SNO464 plus SNO2550 (5ʹ), and SNO467 plus SNO2549 (3ʹ).

#### Tetraploid mating products

Tetraploids [1111,2222], [1111,1111], and [2222,2222] were generated in several steps. First, an allele of the Mating Type-Like Locus (*MTL*) was disrupted in [11,22], [22,22], and [11,11] diploid strains using plasmid pJD1 [42], which indiscriminately replaces either the *MTL*a or *MTL*α locus with the *C*.*d*.*ARG4* gene. From these transformants, *MTL*a cells were identified (i.e., disruptants of the *MTL*α allele), with correct deletion verified by colony PCR using the primers SNO2358–59 (*MTL*a) and SNO2360–61 (*MTL*α). In parallel, the *MTL*a locus was deleted in the same starting strains using fusion PCR to replace the *MTL*a locus with *C*.*d*.*HIS1* (or *C*.*m*. *LEU2* in the H2A.2-FLAG strain); correct transformants were identified by colony PCR. *MT*La/Δ and *MTL*α/Δ strains were switched to the opaque phase and then mated together on YEPD agar medium for 8 hours. Mating products were selected on SD-Arg-His or SD-Arg-Leu medium. Ploidy of the tetraploid mating products was verified by flow cytometry.

#### Epitope tagging of H2A.2

A strain containing a C-terminal fusion of *H2A*.*2* to the 6His/FLAG epitope was created using primers SNO2731–2732 and the pADH52 [36] template to amplify a *FLP-SAT*-marked PCR product containing the epitope and sequences flanking the *H2A*.*2* stop codon. The PCR products was transformed into SN152, and correct integration was verified by colony PCR using the primers SNO2549 plus SN2288, and SNO2550 plus SNO2289. The *FLP-SAT* cassette was then excised by growth in 2% maltose, and correct excision verified by PCR using the primers SNO2549 plus SNO2603.

### Reverse transcription quantitative PCR analyses

RNA was extracted from log or stationary phase cultures grown in YEPD or prespo medium and extracted using a hot acid phenol method, as previously described by Miller and colleagues [12]. RNA was then treated with DNase I (Ambion, Foster City, CA) for 1 hour at 37 °C, followed by a single phenol-chloroform (Ambion, Foster City, CA) extraction to remove DNase activity. A total of 1 μg of RNA was then reverse transcribed into cDNA using SuperScriptIII reverse transcriptase (Invitrogen, Carlsbad, CA) and random hexamer primers. cDNA was quantified using primers to target genes (SNO2735–2736 for H2A.1, SNO3009–3010 for H2A.2), with relative levels normalized to that of the *ACT1* gene (SNO819–820).

### Chromosome loss assays

Chromosome stability was monitored in 3 ways. First, loss of the *ARG4* allele (on Chromosome 7) was determined by measuring the rate of loss of the ability of an *ARG4/arg*Δ heterozygous strain to grow on SD(-Arg) dropout medium. The *ARG4/arg*Δ heterozygous strains were created by integration of 1 copy of *ARG4* from *C*. *dubliniensis* to the *arg*Δ*/arg*Δ locus of WT [11,22], [11,11], [22,22], *bub1*, and *sgo1* strains. *C*.*d*.*ARG4* gene addback fragments targeted to the *arg*Δ locus were generated by fusion PCR using primers SNO143–147 and pSN69 (*C*.*d*.*ARG4*) template, and correct insertion was verified by colony PCR using primers SNO143 plus SNO263 (5ʹ) and SNO147 plus SNO264 (3ʹ). The resultant Arg^+^ strains were streaked onto prespo agar and propagated at 37 °C for 4 days. Cells were then resuspended in sterile H_2_O and plated for single colonies (approximately 200 per plate) on YEPD agar, with incubation at 30 °C for 2 days. Colonies were replica-plated onto SD-Arg agar and monitored for growth; colonies unable to grow on SD-Arg agar were scored as a positive event for chromosome loss. To elicit higher rates of chromosome loss in diploid strains, the *ARG4/arg4*Δ heterozygous WT, [11,22], [11,11], and [22,22] strains were also propagated in YEPD liquid medium containing 1 μg/mL fluconazole (Sigma-Aldrich, St. Louis, MO) for 3 days at 37 °C, prior to plating as above. Loss of Chromosome 7 in arginine auxotrophs was confirmed using quantitative PCR with primers specific to the left and right arms of Chromosome 1 and Chromosome 7 [43]. Chromosome 1 levels were used as a reference, and relative decrease in the abundance of Chromosome 7 was scored as aneuploidy.

The second method took advantage of the fact that *C*. *albicans* contains loci conferring sensitivity to the monosaccharide sorbose dispersed along Chromosome 5 [44]; exposure to sorbose-containing medium selects for cells that have spontaneously lost 1 copy of this chromosome. *C*. *albicans* WT and mutant strains were grown on prespo agar for 4 days at 37 °C, then resuspended in sterile H_2_O; 10^5^ or 10^6^ cells were plated on 2% sorbose (Spectrum Chemical, New Brunswick, NJ)-containing medium, and a dilution was plated on YEPD medium to verify the total number of plated cells. Colonies on YEPD were counted after 2 days at 30 °C, and colonies on sorbose-containing plates were counted after 10 days at 37 °C. Chromosome 5 loss frequencies were calculated as the ratio of colonies observed on sorbose divided by the number on YEPD.

The third method was based on a previously described technique to visualize specific chromosomes using a chromosomally integrated TetO-tandem array and a TetR-GFP fusion protein [45]. In this case, we used a strain (QMY85) in which approximately 120 tandem copies of TetO were integrated into the *his1*Δ locus of *C*. *albicans* reference strain SN87, under selection for the linked *C*.*d*. *HIS1* gene. This strain also contains a codon-optimized TetR-YFP fusion, expressed using an *SNU114* promoter and *ADH1* terminator, under selection for the linked *C*. *maltosa LEU2* gene. Association of the TetR-YFP fusion with the TetO array results in the appearance of 1 yellow fluorescent dot in yeast nuclei. A deletion mutation in the *H2A*.*2* ORF was then introduced into this strain using pSN383 as described above. Samples were analyzed by microscopy as described in the Fluorescence microscopy section.

### Phylogenetic analyses

Sequences encoding H2A-predicted amino acid sequences were individually obtained from NCBI (https://www.ncbi.nlm.nih.gov/) and aligned using a MUSCLE multiple sequence alignment [46]. Multiple sequence alignments were visualized using the ESPRINT program [47]. The phylogenetic tree was generated using a neighbor joining method and visualized using the interactive tree of life online software [48]. The distance measure represents substitutions per amino acid.

### Western blot

Cell numbers were normalized based on OD_600_ and lysed by addition of HU buffer (8 M urea [Millipore, Burlington, MA], 5% SDS [Sigma-Aldrich, St. Louis, MO], 200 mM Tris-HCl [pH 6.8] [Sigma-Aldrich, St. Louis, MO], 1.5% DTT [Sigma-Aldrich, St. Louis, MO]), followed by boiling for 20 minutes. Samples were separated on 12.5% acrylamide gels at 120 V for 30 minutes and blotted onto nitrocellulose membranes (Thermo Fisher Scientific, Waltham, MA). Blotting was performed using a BioRad apparatus in Electroblot Buffer (27.5 mM Tris-Base [Sigma-Aldrich, St. Louis, MO], 192 mM glycine [BioRad, Hercules, CA], 20% methanol [Sigma-Aldrich, St. Louis, MO]) at 20 V for 15 minutes. A primary mouse anti-FLAG antibody (1:500 dilution; Santa Cruz Biotechnology) and a fluorescently labelled anti-mouse secondary antibody (IRDye800CW Goat anti-Mouse IgG; LI-COR, Lincoln, NE) were used to detect H2A.2-FLAG. The primary antibody to H3 was a rabbit anti-H3 (1:5000 dilution; Abcam, Cambridge, United Kingdom) and was detected using a fluorescently labelled anti-goat secondary antibody (IRDye680 Goat anti-Rabbit IgG; LI-COR, Lincoln, NE). Images were acquired using an Odyssey CLx Imaging system (LI-COR, Lincoln, NE).

### Ploidy analyses by PCR

Verification of *H2A*.*1*, *H2A*.*2*, *H2B*.*1*, and *H2B*.*2* gene dosage was performed by qPCR using the following primers: SNO2735–2736 for *H2A*.*1*, SNO3009–3010 for *H2A*.*2*, SNO2725–2726 for *H2B*.*1*, and SNO2729–2730 for *H2B*.*2*, with levels normalized to that of *ACT1* (SNO819–820), which is located separately on Chromosome 5. DNA was purified by phenol-chloroform extraction, and 8 ng was used as a template for qPCR reactions.

For whole-genome ploidy analyses, an established primer set [43] was used, with primer pairs targeting either the left or right arm of each chromosome. For all chromosomes, the primers targeting the left arm of each chromosome were used, with the exception of Chromosome 3, in which both the left and right arm sets were used to confirm whole chromosome duplication. Using these primers, qPCR was performed using 8 ng of DNA template per reaction, and ploidy values were normalized to DNA from the strain SN152, a confirmed diploid control.

### Fluorescence microscopy

*C*. *albicans* was grown in 30 ml cultures at 30 °C with shaking at 200 r.p.m. for 4 to 5 hours in YEPD to an OD_600_ of approximately 1.0. Cells were then harvested by centrifugation, washed 2 times with PBS, spotted onto poly-L-lysine coated coverslips, and allowed to dry. Cells were then fixed with 4% paraformaldehyde (PFA; Electron Microscopy Sciences, Hatfield, PA) for 15 minutes and washed 2 times with phosphate-buffered saline (PBS; UCSF Cell Culture Facility, San Francisco, CA). Vectashield (Vector Laboratories, Burlingame, CA) was applied, followed by application and sealing of a cover slip. Images were acquired under 100× oil immersion objective using a Nikon TiE inverted microscope with Tokogawa spinning disk CSU-X1 using detection settings for YFP (excitation 514 nm, emission 527 nm) and DAPI (Sigma-Aldrich, St. Louis, MO). All images were processed with the ImageJ software (National Institute of Health); the presence of chromosomal YFP spots was counted manually, and >900 cells were evaluated per strain. Only unbudded cells were included in these counts.

### Propidium iodide staining and flow cytometry

Propidium iodide staining and flow cytometry were performed as previously described by Hickman and colleagues [49] with some modifications. Briefly, mid-log phase cells (OD approximately 2.0) were collected and washed 2 times with TE (50 mM Tris base [pH 8] [Sigma-Aldrich, St. Louis, MO], 50 mM EDTA [Sigma-Aldrich, St. Louis, MO]). Cells were pelleted and then fixed in 95% ethanol for 4 hours. Following 2 washes with TE, cells were treated with 1 mg/ml RNase A (Sigma-Aldrich, St. Louis, MO) for 3 hours and then 1 mg/ml proteinase K (Sigma-Aldrich, St. Louis, MO) for 15 minutes. Following 2 washes with TE, cells were resuspended in 50 μg/ml propidium iodide and incubated overnight at 4 °C. Stained cells (200 μl) were diluted in 500 μl of TE and analyzed using an LSR/Fortessa/X-20. Ploidy values were estimated by comparing the ratio of peak locations in experimental samples to that of the diploid control SN152.

### Nocodazole and fluconazole sensitivity assays

WT, [11,22], [11,11], [22,22], *bub1*, *sgo1*, *h2a*.*2* [11,--], *h2a*.*2* plus *H2A*.*2*^WT^, and *h2a*.*2* plus *H2A*.*2*^S121A^ were diluted from overnight cultures and grown to log phase (OD approximately 1.0) in a 30 °C shaker at 200 rpm in YEPD. Cells were then serially diluted onto YEPD, YEPD plus 50 μM nocodazole (Sigma-Aldrich, St. Louis, MO), or 10 μg/ml fluconazole (Sigma-Aldrich, St. Louis, MO), and grown for 24 hours at 30 °C. Plates were imaged using a Canon EOS Revel T5i camera, and image manipulations were performed using the ImageJ software (NIH).

### Statistical analyses

Except for ChIP-seq analysis described below, *p*-values were generated by performing one-way ANOVA with Dunnett’s multiple corrections. Fisher exact test was used to evaluate the significance of the “two dots” comparison. *p* < 0.05 was used as a cutoff for significance.

### ChIP-seq

ChIP was conducted as previously described by Lohse and colleagues [50] with minor alterations. A total of 40 μL of Protein G DYNA beads (Thermo Fisher Scientific, Waltham, MA) were used in place of Sepharose beads for immunoprecipitation, and washes were conducted on a magnetic stand. Post proteinase K treatment, samples were cleaned using Macherey-Nagel PCR clean up columns (Macherey-Nagel, Duren, Germany) and eluted in 36 μL of water, and libraries were made from these samples.

Immunoprecipitations were conducted with 10 μg anti-FLAG M2 antibody (Sigma-Aldrich, St. Louis, MO; cat. F3165), 10 μg anti-histone H3 antibody (Abcam, Cambridge, United Kingdom; cat. ab1791), or 4 μg of anti-Cse4 (Laura Burrack). For all ChIP-seq experiments, cells were propagated in YEPD overnight and seeded the next day at OD_600_ = 0.1 in YEPD, prespo media, or YEPD plus fluconazole (10μg/ml). Samples were propagated to OD_600_ = 1.0 prior to harvesting.

Sequencing library preparation was conducted as previously described by Dumesic and colleagues [51]. Library construction was performed in 2 replicates for each ChIP sample and 1 whole-cell extract (WCE) for each strain utilized. ChIP-seq samples were normalized to their WCEs and viewed on the IGV genome browser.

### ChIP-seq alignment

ChIP-seq data from a HiSeq 4000 were aligned to the *C*. *albicans* genome using Bowtie [1] with the following settings: *bowtie -p[43] -v2 -M1 --best --un {1}_multi_un*.*fastq --max {1}_multi*.*fastq {2} -q [51] --sam {1}_multi*.*sam*

Conversion to BAM files, indexing, and sorting were performed with SAMTools [2].

### Generation of bedgraph files

Bedgraphs were created using BEDTools [3]. Bedgraph files were first expanded to include each position in the genome and then smoothed using a rolling mean of 500 base pairs using the Python package pandas (rolling.mean function). Finally, each position in the bedgraph was normalized to the same position in the smoothed WCE bedgraph file to visualize fold enrichment.

### Whole-genome tile analysis

Each chromosome in the *C*. *albicans* genome was fragmented into 5 kb tiles, and the number of reads aligning within a tile was determined using pysam (https://github.com/pysam-developers/pysam). The RPKM of each tile was calculated by normalizing to the length of the tile in kb and the total number of aligned reads in each sample (reads_T_) as follows:

$${RPKM}_{i}=\frac{{reads}_{i}/{length}_{i}}{{reads}_{T}*1X10^{-6}}.$$

The enrichment (E) of read density in each tile in the ChIP samples over WCE was calculated by dividing the read density from the ChIP sample by the read density from the WCE for each tile:

$$E_{i}=\frac{{RPKM}_{i}^{ChIP}}{{RPKM}_{i}^{WCE}}.$$

*p*-Values were determined by Mann-Whitney U test using the Python package scipy. Raw sequence data and enrichment values for each genome tile are available from GEO (accession number GSE122037).

## Supporting information

S1 Fig Full-length amino acid sequence alignment of core H2A proteins from yeasts and H2A from *Homo sapiens*. S/T121 is denoted by an asterisk. S/T121, serine or threonine at position 121.
(TIF)

S2 Fig Phenotypes and partial genotypes of mutants affecting the *H2A* and *H2B* genes. (A) Colony morphology. Strains were propagated on solid Spider or YEPD medium for 5 days at 30 °C. Please note that *h2a*.*1* and *h2b*.*1*, which contain homozygous deletions of *H2A*.*1* and *H2B*.*1*, respectively, exhibit slow growth on YEPD, whereas the [22,22] strain, which contains extra copies of *H2A*.*2* in place of *H2A*.*1*, grows normally. (B) Genomic copy number of *H2A* and *H2B* genes in *h2A*.*1*, *h2B*.*1*, *h2A*.*2*, and *h2B*.*2* homozygous deletion mutants. Gene abundance was determined by qPCR and normalized to *ACT1* (located on Chromosome 5). (C) Cartoon of the genotype of *h2a*.*1*, with the extra copy of Chromosome 3 highlighted in red. qPCR, quantitative PCR; r.u., relative units; YEPD, yeast extract peptone dextrose.
(TIF)

S3 Fig ORF-swap strains. (A) Cartoon of *H2A* ORF identity in WT, [11,11], and [22,22] diploid strains. (B) Verification of the genomic copy number of *H2A*.*1* and *H2A*.*2* ORFs in these strains, measured by qPCR and normalized to *ACT1*. (C) Verification of *H2A*.*1* and *H2A*.*2* mRNA expression in log-phase strains, measured by RT-qPCR and normalized to *ACT1*. ORF, open reading frame; qPCR, quantitative PCR; RT-qPCR, reverse-transcription quantitative PCR; WT, wild type.
(TIF)

S4 Fig UV-sensitivity of WT and mutant diploid strains after 30 seconds of UV exposure. These data support an additional role for *H2A*.*2* S121 in DNA damage repair. WT, wild type.
(TIF)

S5 Fig Validation of the ploidy of experimental diploid and tetraploid strains. (A) Schematic of WT diploid and WT tetraploid genome content. *C*. *albicans* diploid strains maintain 2 sets of 8 chromosomes (R, 1, 2, 3, 4, 5, 6, and 7), whereas tetraploid strains maintain 4 sets. (B) DNA content of WT diploid and tetraploid strains, as visualized by propidium iodide staining followed by flow cytometry. (C) Colony morphology of WT, WT tetraploid ([1111,2222]), and tetraploids containing only H2A.1 or H2A.2. Strains were propagated for 2 days on YEPD at 30 °C. We note that the H2A.1-containing tetraploid has a more heterogeneous cell size when examined by flow cytometry, and 100% of colonies are highly sectored, consistent with a highly unstable genome. WT, wild type; YEPD, yeast extract peptone dextrose.
(TIF)

S6 Fig ChIP-seq analysis. Shown are plots of bedgraphs of normalized read densities (normalized to the WCE of the sample used for ChIP; see Materials and methods) for the indicated genotypes and conditions across all chromosomes for the same replicate shown in Fig 4. ChIP-seq, Chromatin-immunprecipitation sequencing; WCE, whole-cell extract.
(TIF)

S7 Fig Replicate ChIP-seq data plots. Shown are the normalized enrichments (log2) relative to the WCE sample for 5-kb tiles spanning the *C*. *albicans* genome for replicate experiments performed for the indicated genotypes (SN152: diploid; SN1588: tetraploid) and the indicated conditions. ChIP-seq, Chromatin-immunprecipitation sequencing; Fluc, fluconazole treatment; PreSpo, presporulation medium treatment; WCE, whole-cell extract.
(TIF)

S1 Table Whole-genome ploidy analysis of WT and H2A and H2B homozygous gene disruption mutants. qPCR and primers specific to each *C*. *albicans* chromosome were used to quantify the chromosome abundance in the indicated diploid strains. qPCR, quantitative PCR; WT, wild type.
(XLSX)

S2 Table Assessment of arginine auxotrophy versus loss of Chromosome 7. qPCR and primers specific to *C*. *albicans* Chromosomes 1 and 7 were used to quantify chromosome abundance in the indicated diploid strains. Loss of Chromosome 7 was inferred from depletion relative to the Chromosome 1 reference. qPCR, quantitative PCR.
(XLSX)

S3 Table Strains used in this study. (XLSX)

S4 Table Plasmids used in this study. (XLSX)

S5 Table Primers used in this study. (XLSX)

S1 Data Raw data used for plots in Figs 2B–2D, 2F, 3B–3D and 4A. (XLSX)

## Abbreviations

ANOVA: analysis of variance
bp: base pair
CENP-A: Centromere Protein A
ChIP-seq: chromatin immunoprecipitation with massively parallel DNA sequencing
CPC: Chromosomal Passenger Complex
Cse4: Centromeric Histone H3-like Protein
Fluc: fluconazole
GEO: Gene Expression Omnibus
H2A: Histone 2A
*MTL*: Mating Type-Like Locus
n.s.: nonsignificant
NatR: nourseothricin-resistant
NCBI: National Center For Biotechnology Information
ORF: open reading frame
PFA: paraformaldehyde
PP2A: Protein Phosphatase 2A
prespo: presporulation
qPCR: quantitative polymerase chain reaction
r.u.: relative units
RT-qPCR: reverse transcription with quantitative polymerase chain reaction
S/T121: serine or threonine at position 121
SC-Arg: synthetic complete lacking arginine
Sgo1: Shugoshin 1
TetR: Tetracycline Repressor Protein
WCE: whole-cell extract
WT: wild type
YEPD: yeast extract peptone dextrose
YFP: Yellow Fluorescent Protein
