# Supplementary figures and images for: A natural histone H2A variant lacking the Bub1 phosphorylation site and regulated depletion of centromeric histone CENP-A foster evolvability in Candida albicans
Source: PLoS Biol. 2019 Jun 21;17(6):e3000331. doi: 10.1371/journal.pbio.3000331 (PMC6613695; doi:10.1371/journal.pbio.3000331)

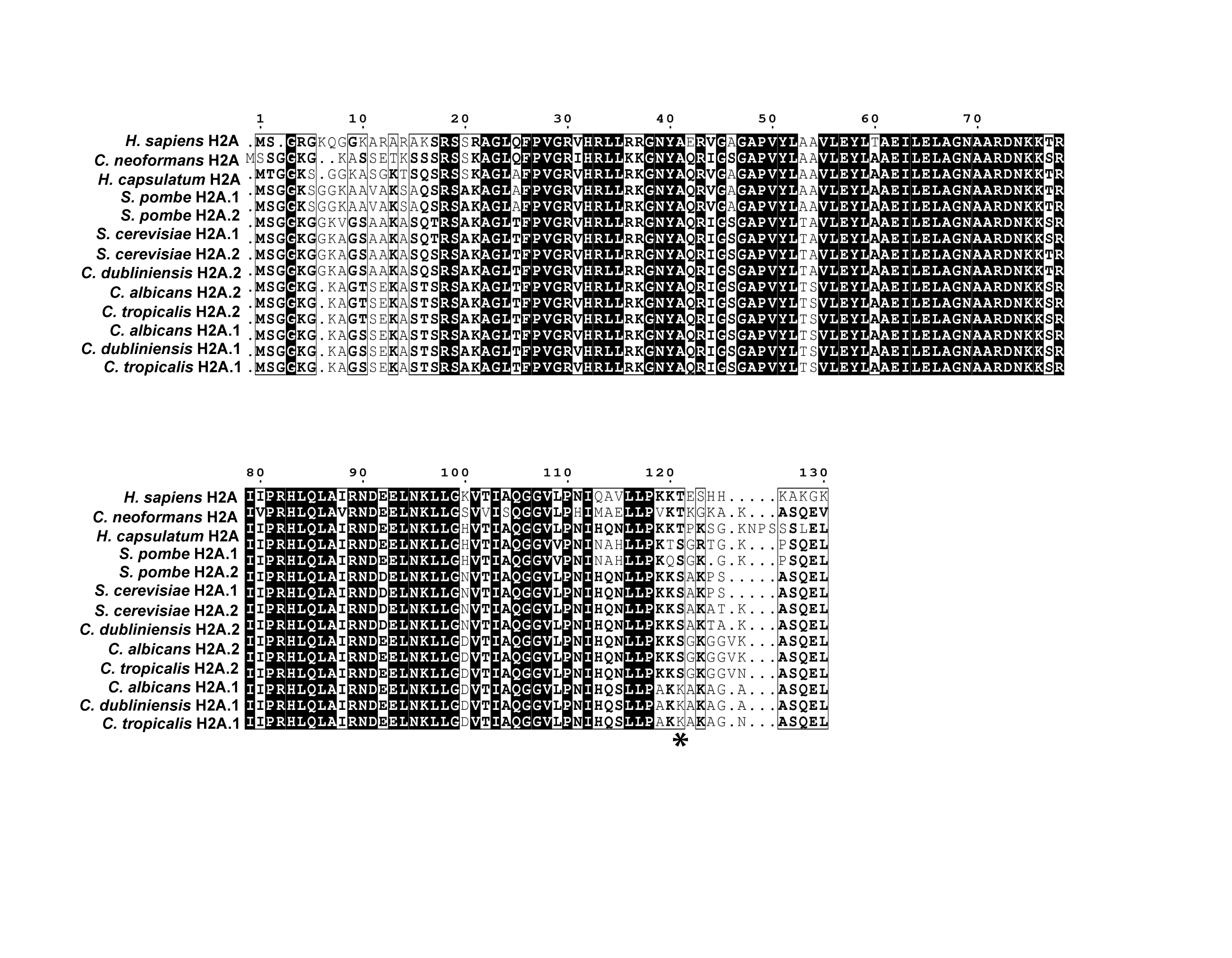

Supplement: S1 Fig — S/T121 is denoted by an asterisk. S/T121, serine or threonine at position 121. (TIF) [file pbio.3000331.s001.tif]

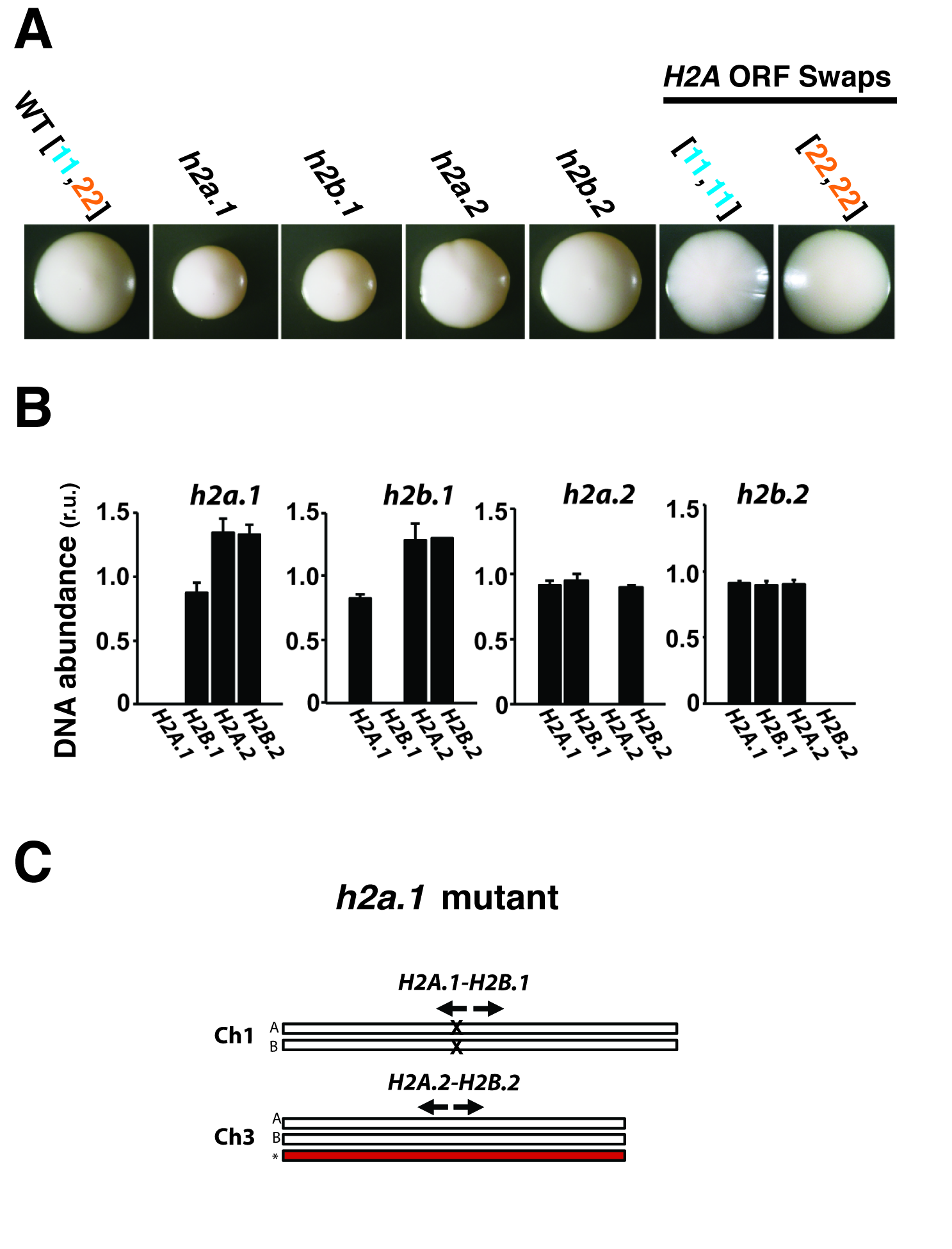

Supplement: S2 Fig — (A) Colony morphology. Strains were propagated on solid Spider or YEPD medium for 5 days at 30 °C. Please note that h2a.1 and h2b.1, which contain homozygous deletions of H2A.1 and H2B.1, respectively, exhibit slow growth on YEPD, whereas the [22,22] strain, which contains extra copies of H2A.2 in place of H2A.1, grows normally. (B) Genomic copy number of H2A and H2B genes in h2A.1, h2B.1, h2A.2, and h2B.2 homozygous deletion mutants. Gene abundance was determined by qPCR and normalized to ACT1 (located on Chromosome 5). (C) Cartoon of the genotype of h2a.1, with the extra copy of Chromosome 3 highlighted in red. qPCR, quantitative PCR; r.u., relative units; YEPD, yeast extract peptone dextrose. (TIF) [file pbio.3000331.s002.tif]

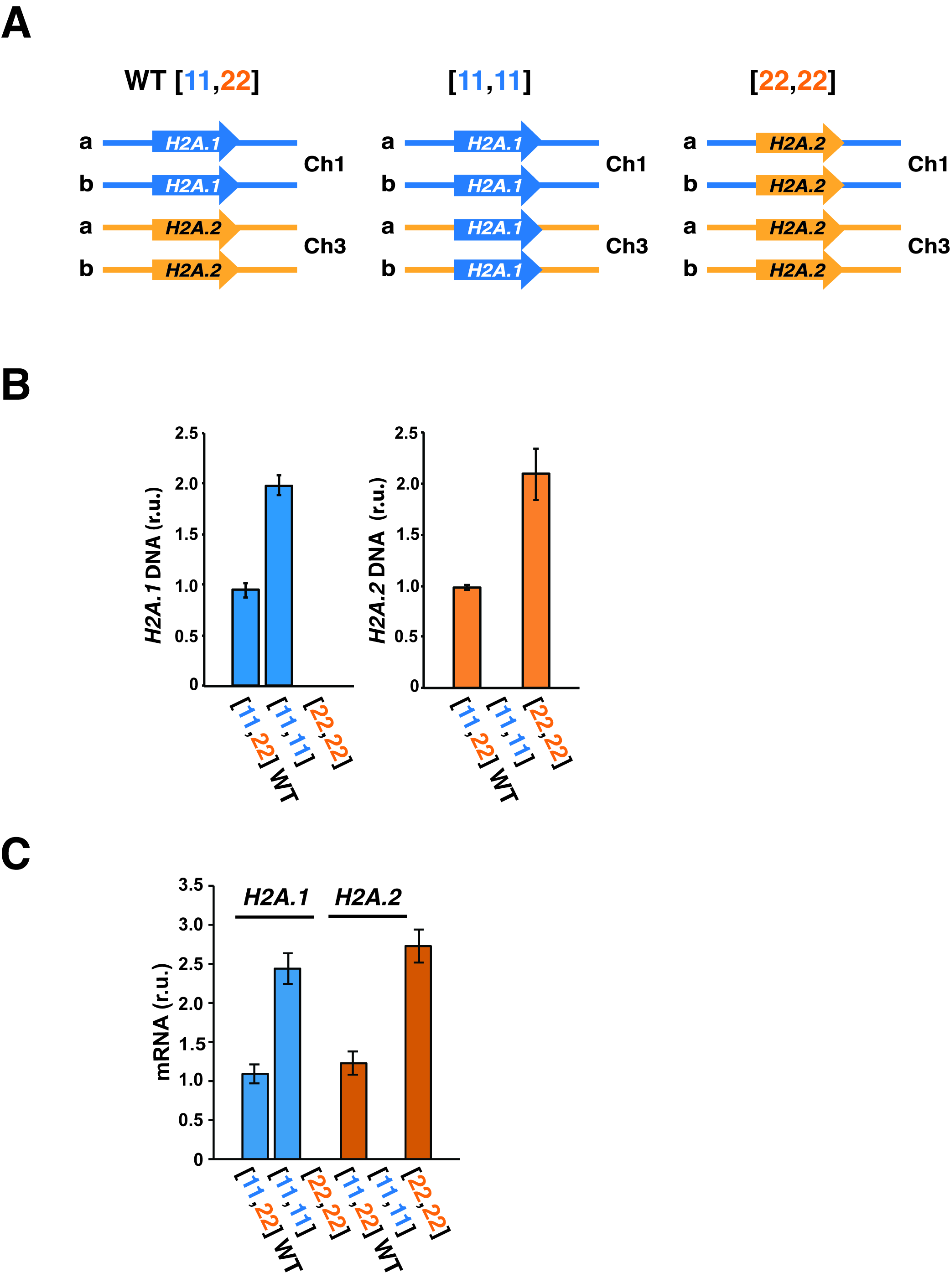

Supplement: S3 Fig — (A) Cartoon of H2A ORF identity in WT, [11,11], and [22,22] diploid strains. (B) Verification of the genomic copy number of H2A.1 and H2A.2 ORFs in these strains, measured by qPCR and normalized to ACT1. (C) Verification of H2A.1 and H2A.2 mRNA expression in log-phase strains, measured by RT-qPCR and normalized to ACT1. ORF, open reading frame; qPCR, quantitative PCR; RT-qPCR, reverse-transcription quantitative PCR; WT, wild type. (TIF) [file pbio.3000331.s003.tif]

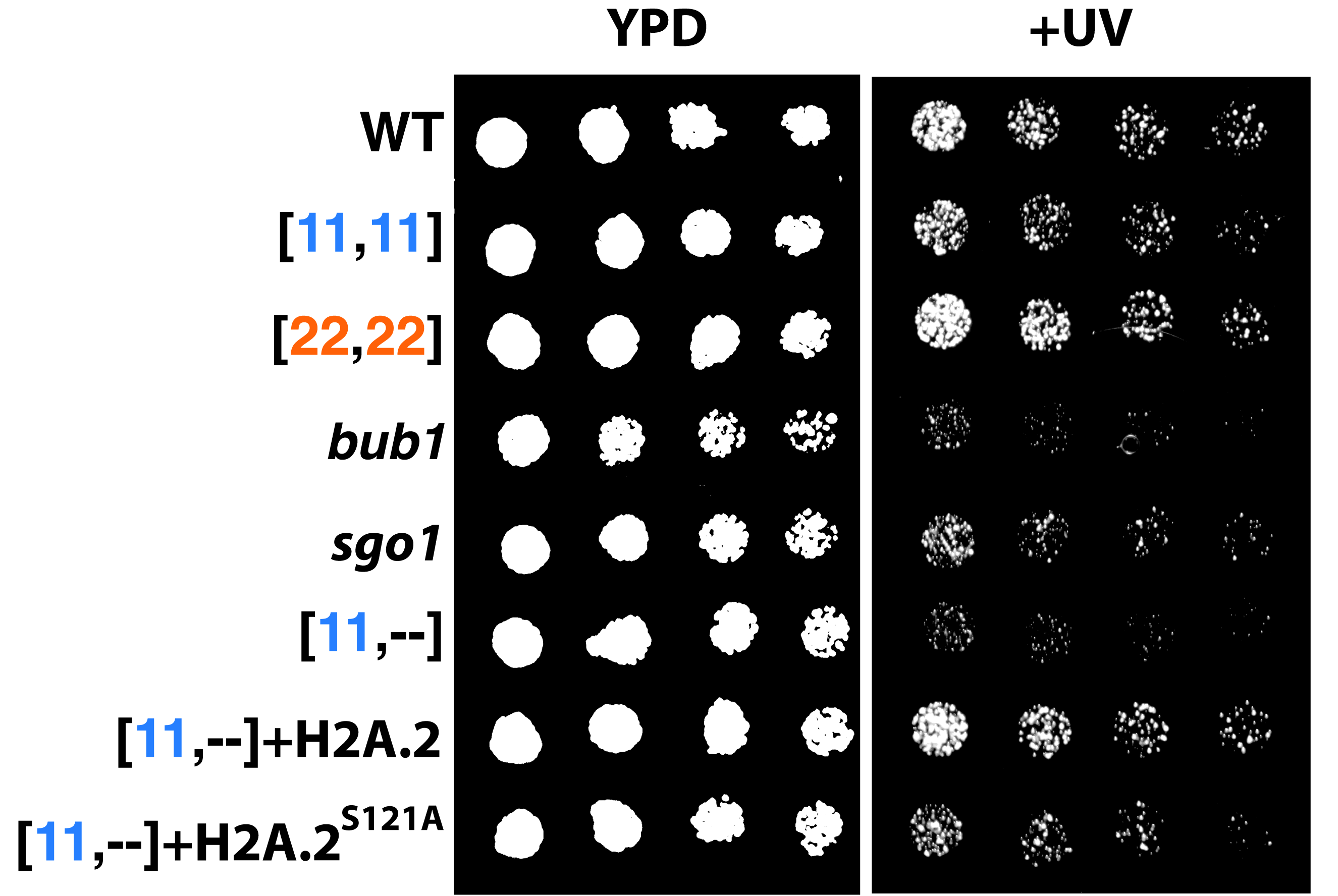

Supplement: S4 Fig — These data support an additional role for H2A.2 S121 in DNA damage repair. WT, wild type. (TIF) [file pbio.3000331.s004.tif]

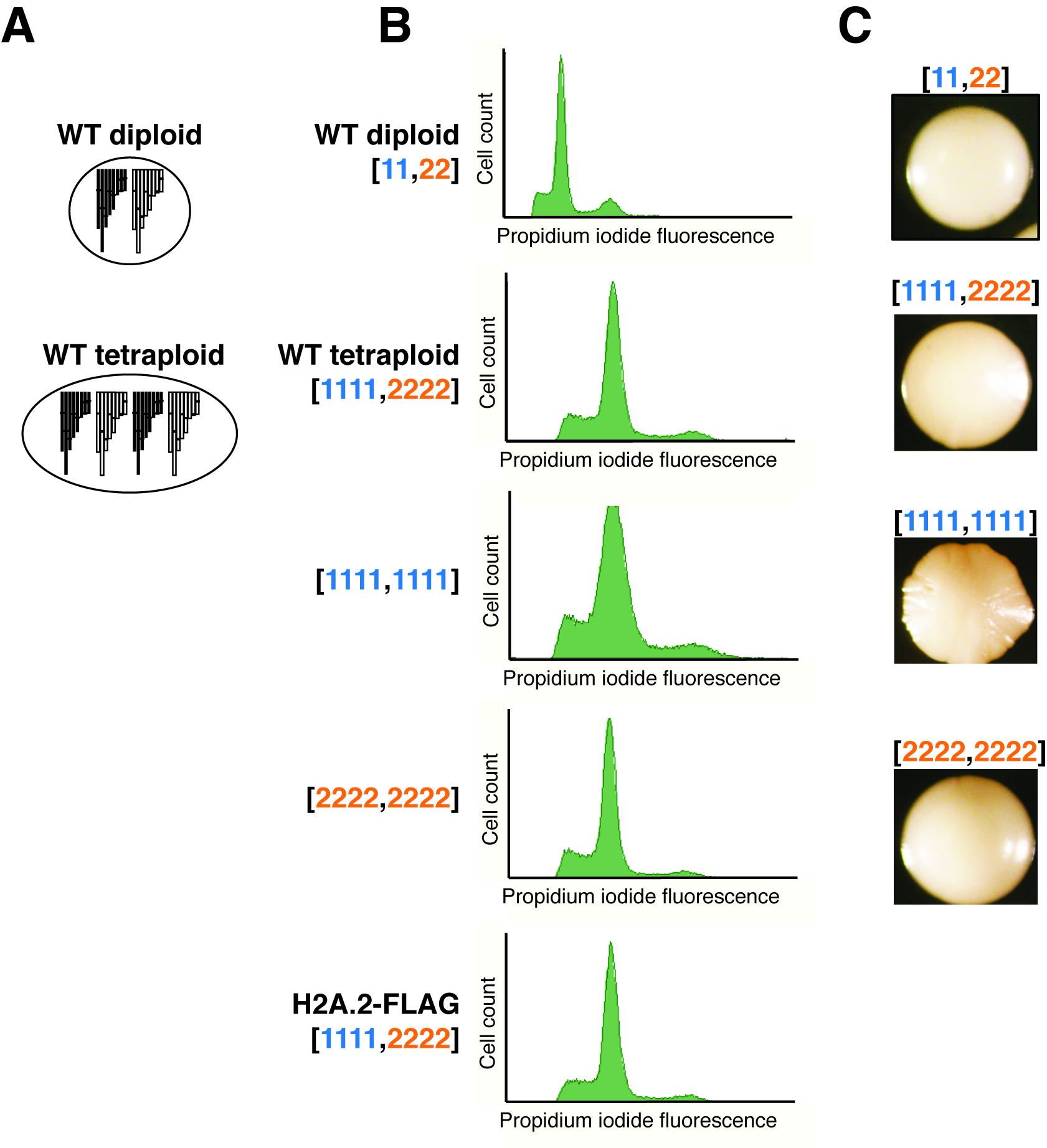

Supplement: S5 Fig — (A) Schematic of WT diploid and WT tetraploid genome content. C. albicans diploid strains maintain 2 sets of 8 chromosomes (R, 1, 2, 3, 4, 5, 6, and 7), whereas tetraploid strains maintain 4 sets. (B) DNA content of WT diploid and tetraploid strains, as visualized by propidium iodide staining followed by flow cytometry. (C) Colony morphology of WT, WT tetraploid ([1111,2222]), and tetraploids containing only H2A.1 or H2A.2. Strains were propagated for 2 days on YEPD at 30 °C. We note that the H2A.1-containing tetraploid has a more heterogeneous cell size when examined by flow cytometry, and 100% of colonies are highly sectored, consistent with a highly unstable genome. WT, wild type; YEPD, yeast extract peptone dextrose. (TIF) [file pbio.3000331.s005.tif]

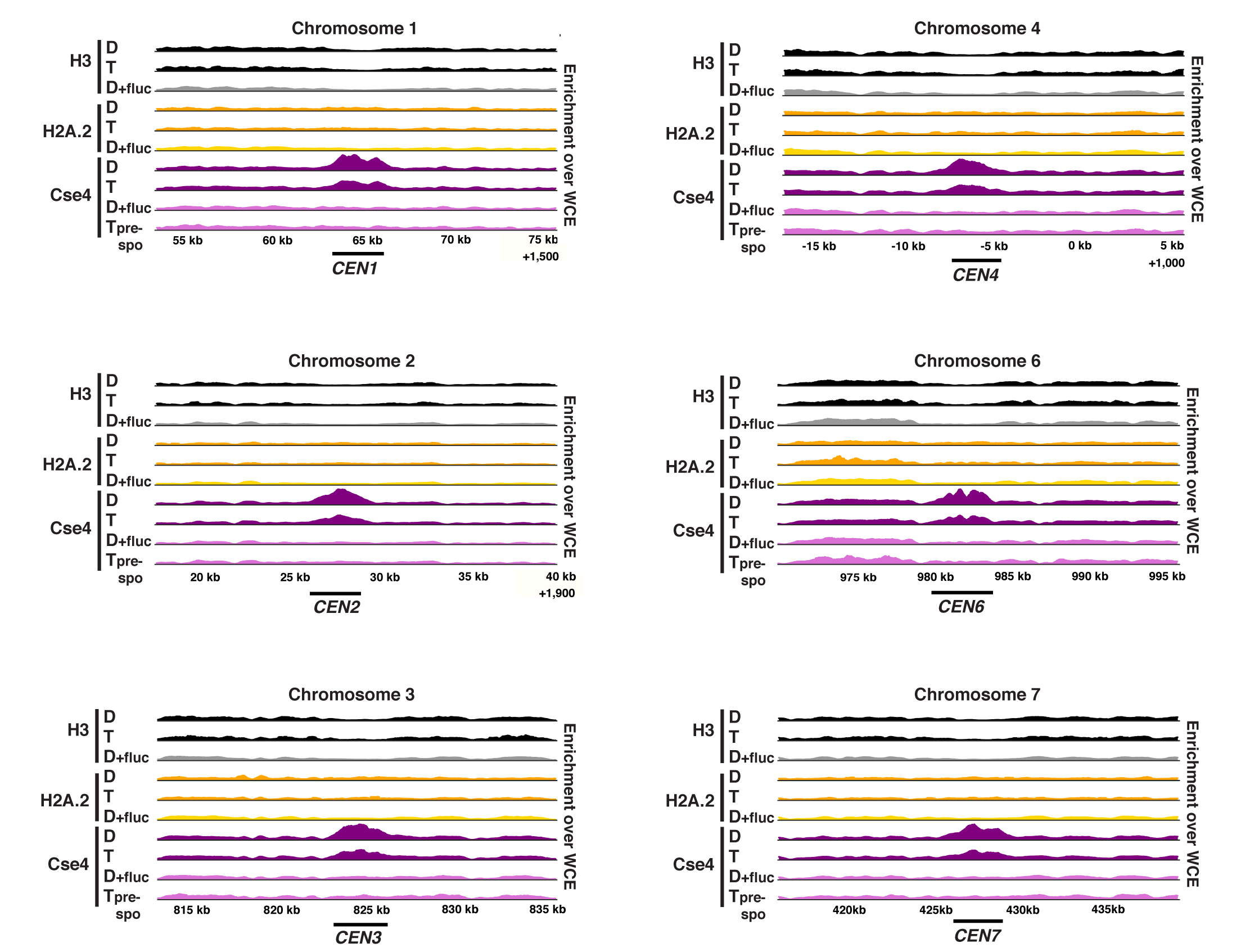

Supplement: S6 Fig — Shown are plots of bedgraphs of normalized read densities (normalized to the WCE of the sample used for ChIP; see Materials and methods) for the indicated genotypes and conditions across all chromosomes for the same replicate shown in Fig 4. ChIP-seq, Chromatin-immunprecipitation sequencing; WCE, whole-cell extract. (TIF) [file pbio.3000331.s006.tif]

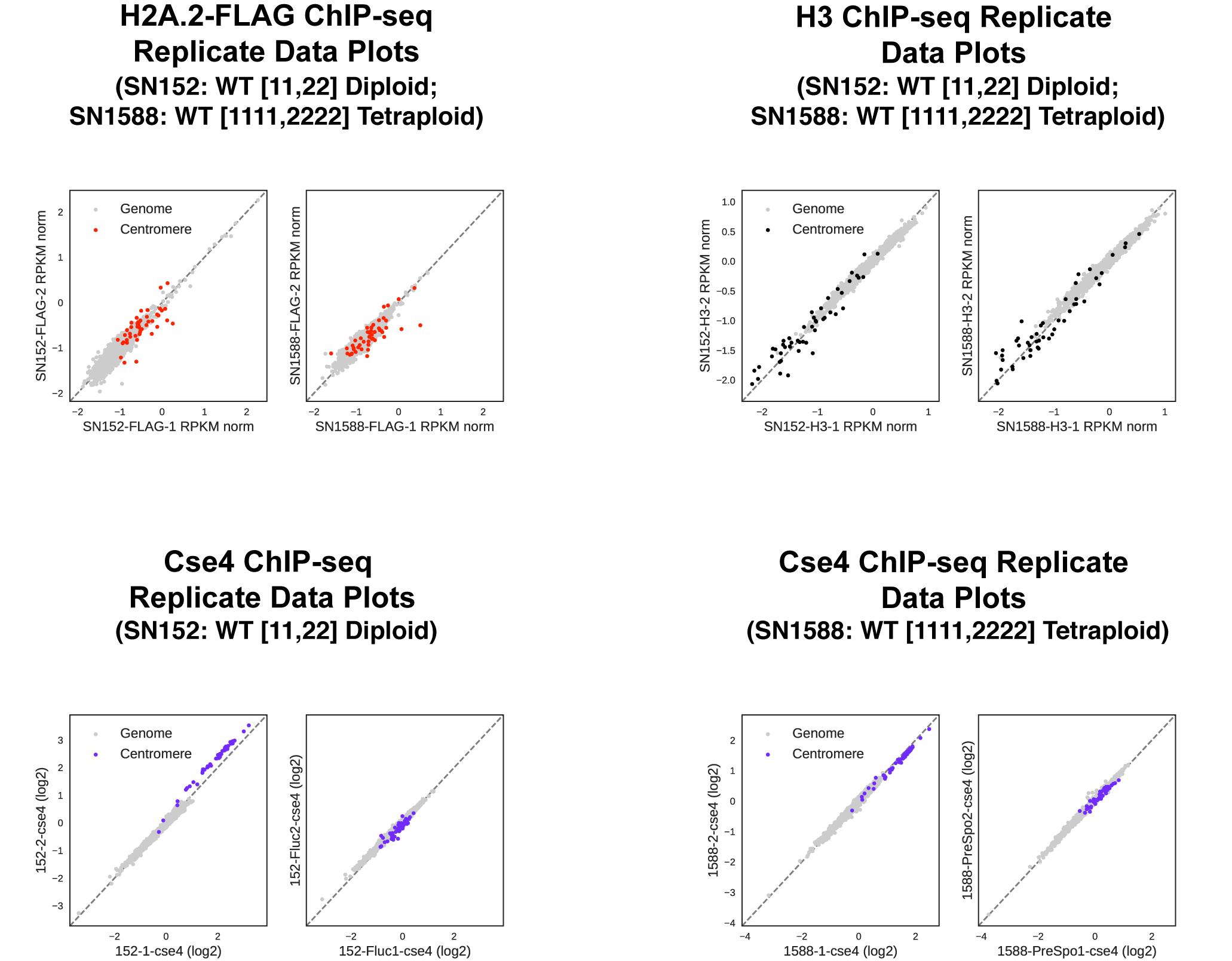

Supplement: S7 Fig — Shown are the normalized enrichments (log2) relative to the WCE sample for 5-kb tiles spanning the C. albicans genome for replicate experiments performed for the indicated genotypes (SN152: diploid; SN1588: tetraploid) and the indicated conditions. ChIP-seq, Chromatin-immunprecipitation sequencing; Fluc, fluconazole treatment; PreSpo, presporulation medium treatment; WCE, whole-cell extract. (TIF) [file pbio.3000331.s007.tif]
